# Designing Co–N/C Cathode Catalysts with Dense Atomic Cobalt Sites for Enhanced PEMFC Performance

**DOI:** 10.1002/advs.202516060

**Published:** 2025-10-15

**Authors:** Mengjun Gong, Asad Mehmood, Ana Guilherme Buzanich, Tim‐Patrick Fellinger, Colleen Jackson, Junyi Cui, Goran Drazic, Anthony Kucernak

**Affiliations:** ^1^ Department of Chemistry, Imperial College London, Molecular Sciences Research Hub White City Campus London W12 0BZ UK; ^2^ Division 3.6, Electrochemical Energy Materials Bundesanstalt für Materialforschung und ‐prüfung (BAM) Unter den Eichen 44–46 12203 Berlin Germany; ^3^ Division for Structure Analysis Bundesanstalt für Materialforschung und ‐prüfung (BAM) Richard‐Willstätter‐Strasse 11 12489 Berlin Germany; ^4^ Department of Chemical Engineering, Imperial College London South Kensington Campus London SW7 2BZ UK; ^5^ Department of Materials Chemistry National Institute of Chemistry Hajdrihova 19 Ljubljana SI‐1000 Slovenia

**Keywords:** non‐precious single atom catalyst, oxygen reduction reaction, proton‐exchange membrane fuel cell

## Abstract

Metal‐nitrogen/carbon (M‐N/C) catalysts, particularly those incorporating Fe, Co, or Mn, are among the most promising non‐platinum group catalysts for the acidic oxygen reduction reaction (ORR) in fuel cells. This study reports a Co‐N/C catalyst featuring high (3 wt%) cobalt content exclusively present as atomic sites. Extended X‐ray absorption fine structure analysis confirms a tetrapyridinic Co‐N_4_ coordination environment in the optimized (3.0)Co‐N/C^Δ^ catalyst. The high cobalt loading leads to a significant density of electrochemically accessible active sites, 3.58 × 10^19^ sites g^−1^, quantified via the nitrite stripping method. The catalyst demonstrates excellent ORR activity in a rotating ring‐disk electrode setup, achieving a half‐wave potential (E_1/2_) of 0.76 V at a low loading of 0.2 mg cm^−2^ and a mass activity of 3.5 A g^−1^ at 0.80 V_RHE_. Single‐cell hydrogen‐oxygen PEMFC tests achieve a peak power density exceeding 1.3 W cm^−2^ (iR‐corrected). Under hydrogen‐air condition, the catalyst delivers 0.54 A cm^−2^ at 0.60 V (0.39 W cm^−2^). Despite the intrinsically higher turnover frequency of Fe‐based sites, the optimized (3.0)Co‐N/C^Δ^ catalyst achieves similar fuel cell performance to that of Fe‐N/C, highlighting the critical role of site density in overall activity.

## Graphical abstract

Cobalt‐based platinum‐metal free catalysts can compete with iron‐based materials as catalysts on the cathodes of fuel cells due to their high site density and activity. This discovery highlights the importance of understanding the active sites responsible for the catalytic activity in these non‐precious metal catalysts.

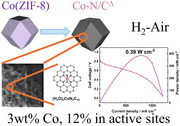

## 1 Introduction

Proton‐exchange membrane fuel cell (PEMFC) is a zero‐emission power generation system that efficiently converts chemical energy to electrical energy by hydrogen oxidation reaction (HOR) and oxygen reduction reaction (ORR) at the anode and cathode sides, respectively. Platinum group metals (PGMs) are generally used as the catalysts for both anodic and cathodic reactions. Approximately 41% of the total cost for fuel cell stacks in light‐duty vehicles^[^ ^1^ ^]^ and a staggering 59% in heavy‐duty vehicles^[^ ^2^ ^]^ can be attributed to the expense associated with PGM application. This substantial cost is among major impediments in the widespread adoption and commercialization of PEMFCs within the transportation sector. The sluggish kinetics of ORR necessitate a larger quantity of PGM catalysts at the cathode side, in contrast to the anode.^[^ ^3^, ^4^ ^]^ Consequently, there is a critical need for alternative ORR catalysts to mitigate this dependency on PGMs. Also, mining and processing of PGMs are associated with large carbon footprints and involve certain supply chain risks, thereby necessitating the need of lowering critical raw material contents and preferably developing sustainable substitutes based on abundant raw materials.^[^ ^5^ ^]^

Non‐precious metal‐nitrogen/carbon catalysts (M‐N/Cs), also called single atom M‐N‐C catalysts, are emerging alternatives to platinum‐based catalysts specifically for ORR at the cathode side. While recent years have seen extensive exploration of various M‐N/C catalysts,^[^ ^6^, ^7^, ^8^, ^9^, ^10^, ^11^, ^12^, ^13^ ^]^ there are only a handful of candidates that are promising for acidic ORR in PEMFCs with notable examples of Fe‐N/Cs,^[^ ^8^, ^14^, ^15^, ^16^, ^17^, ^18^, ^19^, ^20^, ^21^ ^]^ Co‐N/Cs,^[^ ^22^, ^23^ ^]^ Mn‐N/Cs,^[^ ^12^ ^]^ and Sn‐N/Cs.^[^ ^13^ ^]^ While Fe‐N/Cs possess comparatively higher intrinsic activity/turnover frequency (TOF) for ORR, other variants of M‐N/Cs, although less active, are reported to be more stable against degradation owing to either their lower propensity to catalyze Fenton reaction (e.g Co‐N/C and Mn‐N/C)^[^ ^24^, ^25^, ^26^ ^]^ or absence of 2‐electron oxygen reduction pathway that leads to peroxide generation, e.g., Sn‐N/C.^[^ ^13^ ^]^ Thanks to the extensive efforts of multiple research teams, including our own recent breakthrough works, the development of Fe‐N/Cs is comparatively at an advanced stage with ORR activity and single cell PEMFC performance levels approaching those of the Pt‐based counterparts under certain conditions,^[^ ^16^ ^]^ however, that is not the case with the development of non‐iron M‐N/Cs. Most of them still trail significantly behind ORR activities of Fe‐N/Cs in both rotating ring‐disk electrode (RRDE) and single cell configurations. Is this because iron is uniquely favorable as metal in these M‐N/Cs, or can other transition metals achieve similar performance? Boosting ORR activity of non‐iron M‐N/Cs to reach improved PEMFC performance levels requires improvements in active metal loading/active site density (SD) and/or TOFs by careful manipulations of catalyst synthesis strategies.

In our recent work on comparative ORR activity of several different M‐N/C catalysts, Co‐N/C was found to be the most active non‐iron catalyst, although significantly trailing the Fe‐N/C activity, which was due to the combined effects of low Co‐N_4_ site density and inferior TOF.^[^ ^11^ ^]^ Those findings were in line with several other reports in recent literature on the promising ORR activity of Co‐N/Cs for PEMFCs.^[^ ^11^ ^]^ However, as the intrinsic ORR activity/TOF of Co‐N_4_ sites is inferior to that of Fe‐N_4_ sites, substantial enhancement in the density of atomic cobalt sites is essential for Co‐N/Cs to deliver meaningful fuel cell performance levels. A significant challenge toward achieving high metal loadings with atomic dispersion lies in the preparation process to prevent the formation of metal nanoparticles or metal carbides at high temperature, as pyrolysis is inevitable to synthesize M‐N/C.^[^ ^27^, ^28^, ^29^ ^]^ For that reason, currently reported Co‐N/Cs contain only ≈1.0 wt% of atomic Co sites or smaller number of electrochemically active sites even if metal loadings are higher.^[^ ^9^, ^10^, ^30^ ^]^

In this paper, we ask the question: can Co‐N/Cs rival the performance of Fe‐N/Cs? Hence, the focus of this study is to enhance the density of active Co‐N_4_ sites in Co‐N/Cs that can lead to improved single‐cell PEMFC performance. The catalyst synthesis is based on controlled incorporation of cobalt during zeolitic imidazolate framework‐8 (ZIF‐8) preparation, followed by carbonization. ZIF‐8 synthesis is carried out in aqueous medium and is accomplished quickly in half an hour, which is advantageous in the context of sustainability and scalability. Several catalyst samples with varying cobalt content are compared, evaluating the effect of site density on ORR activity as well as comparing their intrinsic activity/TOF with benchmark Fe‐N/C catalysts. Advanced characterization methods, including atomic resolution transmission electron mictroscopy (TEM) and X‐ray absorption spectroscopy (XAS) are employed to confirm the exclusive presence of atomic cobalt sites and understand their coordination environment. Optimized (3.0)Co‐N/C^Δ^ catalyst contains 3.0 wt% Co, which is entirely dispersed as atomic Co‐N_4_ sites and 3.58 × 10^19^ sites g^−1^ as electrochemically accessible sites, which corresponds to a cobalt utilization of 11.7%. The catalytic performance of the best catalyst is promising in both RRDE and PEMFC tests. The half‐wave potential (E_1/2_) is 0.76 V_RHE_ at low loading of 0.2 mg cm^−2^ and a mass activity of 3.5 A g^−1^ at 0.80 V_RHE_. The catalyst delivered an impressive single cell PEMFC performance with peak power densities of above 1.3 W cm^−2^ (iR‐corrected) and 0.39 W cm^−2^ (measured) under H_2_/O_2_ and H_2_/air environments, respectively.

## 2 Results and Discussion

### 2.1 Structural Characterizations of Co‐N/C Catalysts

**Figure 1:**
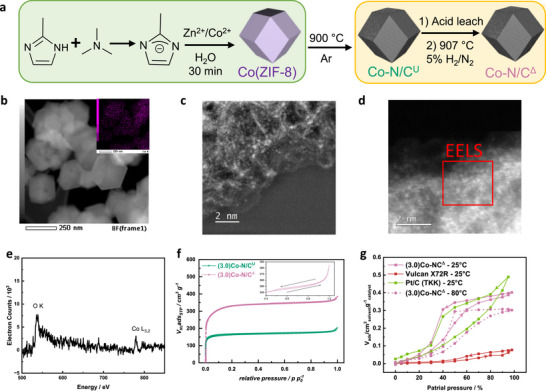
a) schematic Co‐N/C catalyst synthesis. b) HAADF‐STEM image of (3.0)Co‐N/C^Δ^ with inset showing Co elemental mapping by EDXS. c,d) Aberration‐corrected atomic resolution HAADF‐STEM micrographs of (3.0)Co‐N/C^U^ and (3.0)Co‐N/C^Δ^. e) EELS of the framed area of the micrograph in (d). f) nitrogen adsorption‐desorption isotherm of (3.0)Co‐N/C^U^ and (3.0)Co‐N/C^Δ^ g) DVS isotherm using water vapor at 25 °C for (3.0)Co‐N/C^Δ^, Vulcan X72R and Pt/C(TKK TEC10E50E) and at 80 °C for (3.0)Co‐N/C^Δ^.

Cobalt‐doped ZIF‐8 (Co(ZIF‐8)) as a catalyst precursor was synthesized via a 30‐min aqueous synthesis, depicted within the green box in **Figure** **1**a, and the synthesized precursor was analyzed by the X‐ray diffraction (XRD), as shown in Figure S1 (Supporting Information). The XRD peaks of Co(ZIF‐8) matched precisely with those of the commercial ZIF‐8, confirming the successful synthesis of Co(ZIF‐8). Trimethylamine, a weak base, was essential for the successful aqueous synthesis of ZIF‐8 in only 30 min with well‐developed particle morphology. Its function involves deprotonating the 2‐methylimidazole, thereby facilitating the formation of a stable delocalized anion structure, pivotal for ZIF‐8 formation. This anion structure significantly contributes to the successful formation of ZIF‐8, as substantiated by previous literature.^[^ ^31^ ^]^ Co(ZIF‐8) was pyrolysed at 900 °C to obtain (3.0)Co‐N/C^U^, which was then subjected to acid leaching to remove Zn and any unreacted cobalt species, followed by a high‐temperature activation step at 907 °C in a reactive gas environment to obtain the final (3.0)Co‐N/C^Δ^ catalyst. High‐angle annular dark‐field scanning transmission electron microscopy (HAADF‐STEM) of (3.0)Co‐N/C^Δ^ shows polyhedron morphology of primary particles with an average size of ≈250 nm (Figure 1b; Figure S3, Supporting Information). There were no visible variations observed in particle morphology and size between (3.0)Co‐N/C^U^ and (3.0)Co‐N/C^Δ^ catalysts, indicating intact structure of the primary particles during high temperature pyrolysis (Figures S2 and S3, Supporting Information). A particle size in 150–250 nm range is generally desirable for better mass‐transport in catalyst layer of single cell PEMFC.^[^ ^32^, ^33^ ^]^ Particles below 100 nm would hinder the oxygen transport pathway and lower the performance due to high catalyst packing density in the catalyst layer, while larger particles (300–400 nm or above) have a lower external surface area to volume ratio and thus poorer accessibility to the active sites/lower three‐phase interface. Cobalt mapping by energy dispersive X‐ray spectroscopy (EDXS) confirmed a uniform distribution of Co in (3.0)Co‐N/C^Δ^ catalyst (Figure 1b inset). Additional EDXS mapping images of Co and Zn are provided in Figures S2 and S3 (Supporting Information) for (3.0)Co‐N/C^U^ and (3.0)Co‐N/C^Δ^ catalysts, respectively. EDXS mapping reveals significantly lower Zn intensity in (3.0)Co‐N/C^Δ^ catalyst as compared to (3.0)Co‐N/C^U^, confirming the effective removal of Zn from 8.6 to 3.1 wt.% during the activation step, as confirmed by inductively coupled plasma‐mass spectrometry (ICP‐MS) (Table S3, Supporting Information).

Addition of ammonium chloride during the activation step aids Zn removal by producing ZnCl_2_ and ammonia. The ZnCl_2_ can then volatilize (T_vap_(ZnCl_2_) = 732 °C) and the escaping ZnCl_2_ and ammonia may assist in increasing pore access. Aberration‐corrected HAADF‐STEM micrographs of (3.0)Co‐N/C^U^ and (3.0)Co‐N/C^Δ^ shown in Figure 1c,d, reveal isolated bright spots, which signify atomically dispersed Co and Zn, which could not be individually discerned. The electron energy loss spectrum (EELS) of (3.0)Co‐N/C^Δ^ (Figure 1e) indicates a substantial presence of atomically dispersed Co sites in the catalyst. The EELS of carbon in Figure S4 (Supporting Information) show a pre‐peak at ≈285 eV corresponding to the transition to π^*^, indicating sp^2^ character.^[^ ^34^ ^]^ Nevertheless, its relatively low intensity suggests an increase in the degree of sp^3^ defects and a decrease in crystallinity.^[^ ^34^ ^]^ The EELS of (3.0)Co‐N/C^Δ^ suggests the carbon support is amorphous, which is also supported by the Raman spectroscopy (Figure S5, Supporting Information). There are four peaks present in the single atom catalyst, including graphitic peak (G, 1580–1600 cm^−1^), disorder peak (D, 1345–1355 cm^−1^), amorphous carbon peak (A_m_, 1490‐ 1525 cm^−1^), and pentacene peak (P, 1150–1190 cm^−1^).^[^ ^35^, ^36^ ^]^ The crystallite size, L_a_, is inversely proportional to the ratio of the integrated intensity of D and G bands.^[^ ^37^ ^]^ Detailed fitting and calculation methods were discussed in our previously published work.^[^ ^6^ ^]^ The L_a_ of (3.0)Co‐N/C^U^ and (3.0)Co‐N/C^Δ^ are 5.9 ± 0.7 nm and 7.5 ± 0.4 nm, respectively, and the standard deviation was calculated based on 10 fittings at different points in the sample. The increased crystallite size following the second pyrolysis indicates an augmentation in the degree of graphitization.

The nitrogen adsorption‐desorption isotherms of (3.0)Co‐N/C^U^ and (3.0)Co‐N/C^Δ^ show a type I behavior (Figure 1f), suggesting the catalysts are mainly microporous. The apparent specific surface areas (SSAs) are calculated by Brunauer–Emmett–Teller method, and SSAs of (3.0)Co‐N/C^U^ and (3.0)Co‐N/C^Δ^ are 619 and 1235 m^2^ g^−1^, respectively. (Table S1, Supporting Information). The micropore volumes determined by t‐plot are 0.231 and 0.449 cm^3^ g^−1^. Approximate doubling of the surface area and pore volume is attributed to the removal of Zn during the activation step. Also, the SSA of (3.0)Co‐N/C^Δ^ is higher than the transmetalated Co‐N/C catalyst with 0.9wt% Co, (0.9)Co‐N/C(transmet.), prepared from commercial ZIF‐8,^[^ ^11^ ^]^ since the self‐prepared ZIF‐8 has smaller particle size than the commercial one. The cumulative pore volume and pore size distribution are shown in Figure S6 (Supporting Information).

Dynamic vapor sorption (DVS) isotherms of (3.0)Co‐N/C^Δ^, Cabot Vulcan X72R, and Pt/C(TKK TEC10E50E) at 25 °C using water vapor, as well as that of (3.0)Co‐N/C^Δ^ at 80 °C, are shown in Figure 1g. The isotherms of other solvents, including water, isopropanol and n‐heptane, at 25 °C for (3.0)Co‐N/C^Δ^, hot‐pressed (3.0)Co‐N/C^Δ^ based catalyst layer, Vulcan X72R and Pt/C (TKK TEC10E50E) are provided in Figure S8 (Supporting Information). Vulcan X72R demonstrated significantly lower pore volume and increased hydrophobicity as indicated by its much lower water vapor sorption, and delayed onset compared to the other carbon materials. Additionally, the desorption curve closely matched the adsorption, suggesting minimal effects of internal porosity in Vulcan X72R. In contrast, (3.0)Co‐N/C^Δ^ catalyst, exhibited higher water adsorption than Vulcan X72R associated with large interparticle porosity associated with small pore size. A somewhat small hysteresis and levelling off at intermediate water activities point to the (3.0)Co‐N/C^Δ^ catalyst having small hydrophilic pores, which become saturated at lower water partial pressure due to capillary condensation. When water absorption is performed at 80 °C, a temperature more appropriate for fuel cell operation, the onset of water absorption is shifted to higher water activity, and the limiting amount of water absorbed decreases, suggesting that the catalyst becomes more hydrophobic at fuel cell relevant temperatures. This aspect would aid performance under high current/wet conditions as it would allow a significant amount of the pore structure to remain unwetted and hence available for fast gas phase reactant transport. Based on the isotherms for different solvents, the surface tension of catalyst (γ_s_), comprising the dispersive (γ_s_^d^) and polar (γ_s_^p^) components, spreading pressure (π_e_) and work of adhesion (W_s‐l_) were calculated.^[^ ^38^ ^]^ The calculated parameters, displayed in Table S2 (Supporting Information), are compared with commercial Pt/C (TKK TEC10E50E) and Vulcan X72R. The former is the most hydrophilic with Vulcan X72R and (3.0)Co‐N/C^Δ^ having similar surface energies and lower equilibrium spreading pressure, π_e_, than TKK Pt/C. This indicates that adsorption of vapor has little effect on the surface energy of Vulcan X72R and (3.0)Co‐N/C^Δ^, but a much larger variation on Pt/C – probably because of the heterogeneous nature of Pt/C comprising a hydrophobic carbon and hydrophilic metal. The deviations in γ_s_ between Pt/C on the one hand and Vulcan X72R and Co‐N/C on the other are due to the polar components of the surface energy (γ_s_^p^), probably associated with the presence of hydrophilic platinum in the material. Considering absorption of solvents other than water (Figure S7, Supporting Information), although the (3.0)Co‐N/C^Δ^ catalyst can sorb a significant amount of water at relative partial pressures >50%, at 100% water partial pressure ≈20% of the pore volume wetted by the more non‐polar solvents remains unwetted by water and hence are still available for gas transport. In contrast, the response of the Pt/C to different solvents shows that there is no residual pore volume for gas transport at high water partial pressure, suggesting that oxygen access to ORR sites in pores might be hindered due to diffusion through water‐filled pores. This aspect is important to consider for a fuel cell operating at high current densities under humid environments in which flooding of the cathode can lead to significant mass transport problems. The ability of a catalyst to maintain some non‐wetted pores under these conditions allows efficient two‐phase flow within the catalyst layer.

We also provide calculated values of the parameters for CCM/CCLs composed of Pt/C and Co‐N/C in Table S2 (Supporting Information). These values are provided for comparison and should be treated with caution as they are calculated for a heterogeneous system composed of two separate phases, and so the calculated parameters are a weighted average of the multiple interfaces present.

**Figure 2:**
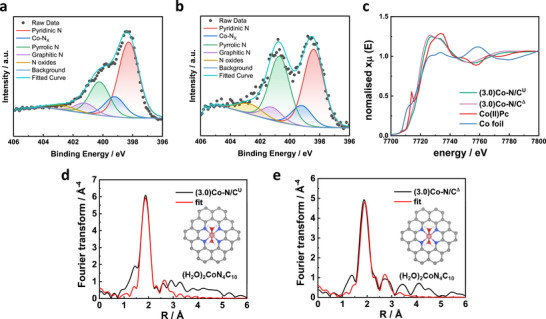
a,b) Deconvoluted high resolution N 1s XPS spectra of (3.0)Co‐N/C^U^ and (3.0)Co‐N/C^Δ^, respectively. c) XANES of (3.0)Co‐N/C^U^, (3.0)Co‐N/C^Δ^ and reference materials, including Co foil and cobalt(II) phthalocyanine. d,e) fitted Co K‐edge EXAFS profiles of (3.0)Co‐N/C^U^ and (3.0)Co‐N/C^Δ^, respectively. Fourier transformed space is shown without a phase‐shift correction. Model structures used for EXAFS fitting are shown in insets.

**Figure 3:**
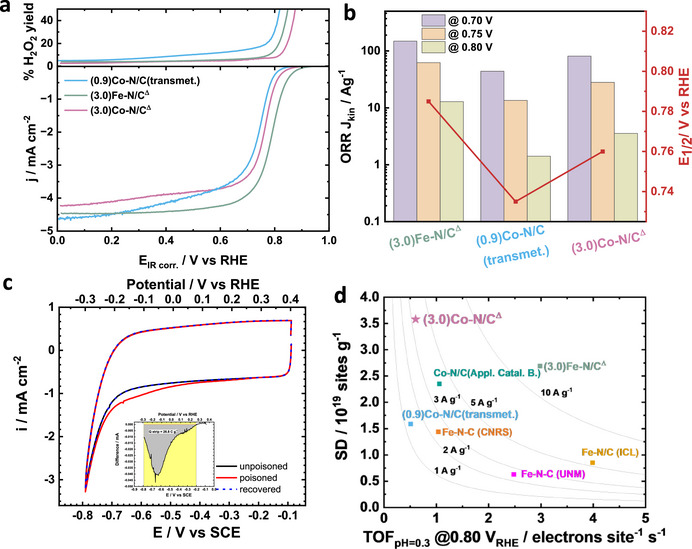
a) ORR polarization curves of (3.0)Co‐N/C^Δ^, (3.0)Fe‐N/C^Δ^, and (0.9)Co‐N/C(transmet.) with a rotation rate of 1600 rpm in O_2_‐saturated 0.5 M H_2_SO_4_ electrolyte. Catalyst loading was 0.2 mg cm^−2^. b) comparison of kinetic mass activity at different potentials and half‐wave potential of the three catalysts. c) nitrite stripping cyclic voltammetry in Ar‐saturated electrolyte of (3.0)Co‐N/C^Δ^. Inset shows excess current associated with the reductive stripping of nitrite. d) isoactivity plot comparing the optimized (3.0)Co‐N/C^Δ^ catalysts with (0.9)Co‐N/C(transmet.), literature‐reported high performance Co‐N/C^9^ and benchmark Fe‐N/C catalysts from our previous work.^[^ ^47^ ^]^ transmet. = transmetallated.

Elemental compositions of the catalyst materials determined by XPS and ICP‐MS are provided in Table S3 (Supporting Information). The optimized (3.0)Co‐N/C^Δ^ catalyst has a cobalt loading of 3.0 wt%, which is 3.3 times greater than the (0.9)Co‐N/C(transmet.) sample that was prepared by Zn‐templating approach using a commercial ZIF‐8^[^ ^11^ ^]^ (Table S3, Supporting Information). Nitrogen plays a crucial role in stabilizing transition metal ions, forming single‐atom catalysts.^[^ ^39^ ^]^ Hence, the nitrogen functionalities are pivotal and were assessed through the deconvolution of high‐resolution N 1s XPS spectra (**Figure** **2**a,b). Five distinct nitrogen peaks, including pyridinic nitrogen (398.5 eV), nitrogen bonded to Co (399.4 eV), pyrrolic nitrogen (400.3 eV), graphitic nitrogen (401.2 eV), and nitrogen oxides (402.7 eV) were used for deconvolution. Relative amounts of different nitrogen sites are given in Table S4 (Supporting Information). A comparison of Figure 3a and b reveals that relative ratio of pyridinic to pyrrolic nitrogen decreases after activation. This is likely due to the removal of Zn which is reduced from 8.6 to 3.1 wt% after activation (Table S3). The nitrogen coordinated with a metal ion (e.g. Zn or Co) often overlaps with the pyridinic nitrogen and is hard to distinguish.^[^ ^40^, ^41^ ^]^ Therefore, the apparent relative decrease in pyridinic nitrogen and relative increase in the pyrrolic nitrogen for (3.0)Co‐N/C^Δ^ sample may be explained by reduced Zn‐N coordination and/or predominant loss of pyridinic nitrogen sites during high temperature activation as the overall nitrogen content is decreased from 17.2 to 5.59 at.% post‐activation. A nitrogen content in 5–6 at.% range is generally desirable in the final activated catalyst as it is sufficient enough to stabilize atomic metal sites via Co‐N_4_ coordination without compromising the electronic conductivity of carbon framework, which is important for operating PEMFC at high current densities.

The coordination environment of atomic Co sites was analyzed by X‐ray absorption spectroscopy (XAS). X‐ray absorption near edge structure (XANES) of (3.0)Co‐N/C^U^, (3.0)Co‐N/C^Δ^, and reference materials are shown in Figure 2c. The pre‐edge peak of Co(II) phthalocyanine is ≈7716 eV, indicating a perfect square planar Co‐N_4_. However, the pre‐edge peaks of (3.0)Co‐N/C^U^ and (3.0)Co‐N/C^Δ^ are around 7708–7710 eV, and the intensities are slightly higher than Co(II) phthalocyanine, which is corresponding to the non‐planar Co‐N4 sites.^[^ ^42^ ^]^ The edge energy for Co(II) is ≈7730 eV according to the literature and also reference material.^[^ ^9^, ^10^, ^42^ ^]^ (3.0)Co‐N/C^U^ and (3.0)Co‐N/C^Δ^ have slightly higher oxidation state than 2+ which can be expected due to the adsorption of axial ligands. The Fourier‐transformed (FT) fitted extended x‐ray absorption fine structure (EXAFS) profiles of (3.0)Co‐N/C^U^ and (3.0)Co‐N/C^Δ^ catalyst are fitted very well with a tetrapyridinic Co‐N_4_ model structure with two water ligands (Figure 2d,e). Therefore, a nitrogen‐doped carbon framework with a higher proportion of pyridinic nitrogen would be favorable, as Co tends to coordinate with pyridinic nitrogen. Density functional theory  (DFT) calculations suggest that the binding energy of pyridinic nitrogen with Co is the lowest among all first‐row transition metals,^[^ ^43^ ^]^ indicating that the formation of a tetrapyridinic Co‐N_4_ is the most energetically favorable configuration. Moreover, some DFT calculations suggest that the amount of pyridinic nitrogen has a greater effect on ORR performance than other types of nitrogen, such as pyrrolic or graphitic nitrogen.^[^ ^44^, ^45^ ^]^ By comparing the free energy of pyridinic M‐N_4_ and pyrrolic M‐N_4_ sites with key intermediates of the ORR mechanism at 0.7 V_RHE_, it was found that pyridinic Co‐N_4_ can stabilize the adsorption of O and OH species, indicating high ORR activity for inner sphere mechanism.^[^ ^46^ ^]^ The corresponding fitted parameters are provided in Table S5 (Supporting Information). For both catalysts, the coordination number (CN) of Co with nitrogen is 4, and the Co–N bond length is ≈1.9 Å. DFT calculations reported in the literature suggest that shorter Co–N bond lengths correspond to more stable Co sites, as the Gibbs free energy for metal leaching from oxygen‐adsorbed sites increases with decreasing bond length, making demetallation less likely.^[^ ^10^ ^]^ Formation of tetrapyridinic Co‐N_4_ sites (CoN_4_C_10_) is more likely as compared to Zn, which forms tertrapyrollic Zn‐N_4_ sites (ZnN_4_C_12_) as predicted by DFT calculations.^[^ ^43^ ^]^ Moreover, there are no indications of Co‐Co nearest neighbors, indicating the absence of any metal nanoparticles formation.

### 2.2 Electrochemical Performance

The oxygen reduction activities of (3.0)Fe‐N/C^Δ^, (0.9)Co‐N/C(transmet.), and (3.0)Co‐N/C^Δ^ were evaluated in RRDE setup in a 0.5 M H_2_SO_4_ electrolyte (pH 0.3), and the results are compared in **Figure** **3**a. Different catalyst preparation conditions, including pyrolysis at higher temperatures and under different atmospheres, were investigated, and the results are shown in Figure S9 (Supporting Information). Pyrolyzing at higher temperatures (e.g., 1050 °C) can be beneficial for removing more Zn and for generating a more conductive graphitic carbon framework. However, it also risks removing nitrogen from the nitrogen‐doped carbon framework, causing Co single atoms to aggregate and thereby diminishing the high ORR activity. In addition, activation under a reducing atmosphere (5% H_2_/N_2_) was found to further improve the performance. An activity comparison with (3.0)Fe‐N/C^Δ^ and (0.9)Co‐N/C, with detailed synthetic methods provided in Note S1 (Supporting Information), was carried out to evaluate the performance of high cobalt loading (3.0)Co‐N/C^Δ^ catalyst in a meaningful way. The transmetalation method was very effective with noteworthy 7.0 wt% Fe as a single‐atom site for Fe‐N/C in our recent work,^[^ ^6^ ^]^ while its effectiveness was found to be moderate toward Co‐N/C synthesis. (3.0)Co‐N/C^Δ^ catalyst with 3 wt% atomic Co sites exhibits excellent ORR activity with an onset potential of 0.85 V_RHE_ (measured at −0.1 mA cm^−2^) that is 30 mV higher than that of the (0.9)Co‐N/C(transmet.), which has 0.9 wt% cobalt loading. (3.0)Fe‐N/C^Δ^ catalyst clearly has the highest ORR activity in high potential region with an onset potential of 0.89 V_RHE_. This observation is in line with the commonly reported higher intrinsic activities/TOFs of Fe sites, however, thanks to the higher loading of Co, the ORR activity gap between Co and Fe catalysts is significantly narrower in this case when compared with the previous reports.^[^ ^11^, ^48^ ^]^ The activity of (3.0)Co‐N/C^Δ^ catalyst is accelerated in 0.75–0.80 V_RHE_ range with an impressive E_1/2_ value of 0.76 V_RHE_ (despite low loading of 0.2 mg cm^−2^), which is 25–30 mV higher than the other Co catalyst (Figure 3b). A comparison of E_1/2_ values with other literature, including Fe‐N/Cs and Co‐N/Cs, is shown in Table S6 (Supporting Information). The ORR performance of (3.0)Co‐N/C^Δ^ was comparable to that of most non‐precious metal single atom catalysts and even superior to some of the Fe‐N/C catalysts with low metal content (< 4 wt%). This also demonstrates that, with a high site density, the performance of Co–N/C catalysts can be comparable to, or even exceed, that of Fe–N/C catalysts. (3.0)Fe‐N/C^Δ^ exhibited very high ORR performance in RDE at high potentials (>0.8 V_RHE_), benefiting from its high intrinsic TOF of Fe‐N_4_. However, (3.0)Co‐N/C^Δ^ showed a very similar kinetic mass activity at 0.7 V_RHE_ owing to its high metal loading and electrochemical accessible sites. In contrast, the (0.9)Co‐N/C(transmet.) catalyst, prepared by transmetalation, exhibited a kinetic mass activity that was two‐fold lower than that of (3.0)Fe‐N/C^Δ^. Notably, an ORR kinetic mass activity value of 3.5 A g^−1^ is obtained at 0.80 V_RHE_ for (3.0)Co‐N/C^Δ^ catalyst which is 2.5 times greater than 1.4 A g^−1^ of (0.9)Co‐N/C(transmet.) (Figure 3b). The method used for calculating kinetic mass activity is detailed in Figure S10 (Supporting Information). While all three catalysts show relatively lower peroxide yields, (3.0)Co‐N/C^Δ^ has the lowest value in the range of 3–4% (Figure 3a).

The stability of (3.0)Co‐N/C^Δ^ catalyst was evaluated by potential cycling in O_2_‐satuated 0.5 M H_2_SO_4_ electrolyte (Figure S10, Supporting Information). The catalyst activity was fairly stable with an E_1/2_ loss of only 19 mV after 5000 cycles, which is significantly smaller than generally observed for Fe‐N/Cs, which usually undergo larger degree of activity degradation during potential cycling in oxygen‐saturated electrolyte.^[^ ^49^, ^50^ ^]^ Interestingly, most of the decay of (3.0)Co‐N/C^Δ^ catalyst activity was observed during the first 3000 cycles with very stable performance in the subsequent 2000 cycles. The change in kinetic mass activities calculated at various voltages before, during, and after the cycling is provided in Figure s10b (Supporting Information).

Quantification of electrochemically accessible active sites in (3.0)Co‐N/C^Δ^ and (3.0)Fe‐N/C^Δ^ catalyst was carried out by in situ nitrite stripping approach, and the corresponding cyclic voltammograms are shown in Figures S11 and S12 (Supporting Information), respectively. A clear nitrite reduction peak is observed between 0.1 and −0.25 V_RHE_ in the poisoned CV at testing a large number of atomic cobalt sites (Figure 3c). The value of stripping charge obtained from the reduction of nitrite peak is 28.6 C g^−1^ (Figure 3c inset) which translates into a SD value of 3.58 × 10^19^ sites g^−1^ corresponding to a utilization of 11.70% of the total Co‐N_4_ sites (Table S7, Supporting Information). To the best of our knowledge, this is the highest value of Co‐N_4_ site density when compared with the reported literature. Although the same molar ratio of Co:Zn and Fe:Zn were used to prepare the (3.0)Co‐N/C^Δ^ and (3.0)Fe‐N/C^Δ^, the numbers of electrochemically accessible sites in (3.0)Fe‐N/C^Δ^ was ≈30% lower than that of (3.0)Co‐N/C^Δ^. Isoactivity plot in Figure 3d provides a comparison of SD and TOF (which was calculated using nitrite‐based site density and ORR kinetic mass activity at 0.80 V_RHE_ measured in 0.5 m H_2_SO_4_ electrolyte) of the (3.0)Co‐N/C^Δ^ catalyst with benchmark Fe‐N/Cs and a Co‐N/C from literature that had a high site density.^[^ ^9^, ^47^ ^]^ From the isoactivity plot, one can clearly observe that Fe‐N/Cs generally have higher TOFs (≥ 2 electrons site^−1^ s^−1^) while Co‐N/Cs have TOF values in lower range between 0.6 and 1.0 electrons sites^−1^ s^−1^. Therefore, increasing the density of active sites for Co‐N/Cs is an effective way to improve overall ORR activity and narrow the performance gap with Fe‐N/Cs. The (3.0)Co‐N/C^Δ^ catalyst with 3wt% cobalt loading has the highest SD among all the catalysts compared and is 3.5 times greater than that of the (0.9)Co‐N/C(transmet.) (Figure 3d). To gain greater insights into the link between cobalt loading and density and activity/TOF of electrochemically active Co sites as well as the effect of Co‐N/C synthesis methods, additional catalyst samples were prepared. (0.8)Co‐N/C^Δ^ catalyst has low cobalt loading of 0.8 wt.% and was prepared exactly the same way as the optimized (3.0)Co‐N/C^Δ^ catalyst. (0.5)Co‐N/C(transmet.) was prepared by transmetalation method and has a cobalt loading of 0.54 wt.%. Synthesis details of these additional catalyst samples are provided in Note S1 (Supporting Information). Cobalt loadings as well as site density values determined by nitrite stripping for all Co‐N/C samples are listed in Table S7 (Supporting Information).

The density of electrochemically accessible sites increased linearly with cobalt loading for the four Co‐N/C catalysts that had cobalt content between 0.5 and 3.0 wt% and were synthesized by two different methods (Figure S13a and Table S7, Supporting Information). This correlation suggests that increasing the Co loading assists in increasing the electrochemical site density and is independent of the catalyst synthesis methods used in this study. Interestingly, the fitted line has a non‐zero y‐intercept of 0.61 × 10^19^ site g^−1^, indicating the presence of some nitrite accessible sites which are not linked to the active metal (Co) but to the ZIF‐8 derived NC framework, as we explained in greater detail in our recent work.^[^ ^6^ ^]^

### 2.3 PEMFC Performance

**Figure 4:**
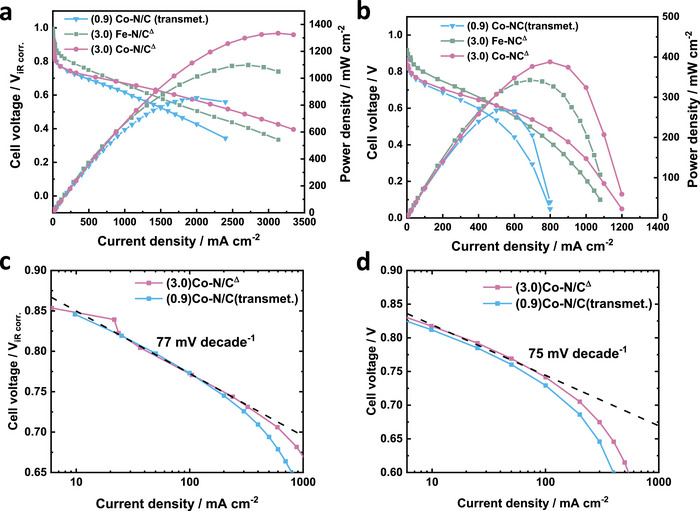
a) H_2_‐O_2_ and b) H_2_‐air polarization curves of single cell PEMF with (3.0)Co‐N/C^Δ^, (3.0)Fe‐N/C^Δ^, and (0.9)Co‐N/C(transmet.) catalysts. c) Tafel plot of H_2_‐O_2_ and d) Tafel plot of H_2_‐air polarization curves. Test conditions: 5 cm^2^ MEA consisting of a 0.4 mg_Pt_ cm^−2^ Pt/C anode, a Co‐N/C catalyst cathode, and a Nafion 211 membrane. Measurements were performed at 80 °C and 100% relative humidity. For H_2_‐O_2_ test, the flow rates were 200 sccm for both sides. For H_2_‐air test, the flow rates of H_2_ and air were 300 and 1000 sccm, respectively. 1 bar gauge pressure on both sides.

Single‐cell PEMFC performance assessment of (3.0)Co‐N/C^Δ^_,_ (3.0)Fe‐N/C^Δ^, and (0.9)Co‐N/C(transmet.) cathode catalysts was carried out using a 5 cm^2^ cell (Notes S1, Supporting Information) under hydrogen‐oxygen and hydrogen‐air conditions, presented in **Figure** **4** and Figures S14 and S15 (Supporting Information). Due to its higher TOF, the (3.0)Fe‐N/C^Δ^ catalyst exhibited superior performance in the high‐potential region and achieved an OCP close to 1.0 V under H_2_‐O_2_ conditions. Notably, under the same testing conditions, (3.0)Co‐N/C^Δ^ exhibits a current density of 50 mA cm^−2^ at 0.80 V_iR‐free_ and reaches a peak power density of 1.33 W cm^−2^, surpassing that of (3.0)Fe‐N/C^Δ^ and showing that under operational conditions (0.6–0.7 V), Co based catalysts can exceed the performance of Fe based catalysts within the same catalyst morphology and structure. Notably, this performance surpasses those of the reported non‐precious metal‐based single‐atom catalysts under similar conditions (Table S8, Supporting Information). Both (3.0)Co‐N/C^Δ^ and (0.9)Co‐N/C(transmet.) catalysts display overlapping current densities in the activation region/high voltage region due to their similar structure suggesting equivalent activities of their active sites (Figure 4a). However, (3.0)Co‐N/C^Δ^ exhibits significantly higher performance at higher current densities >500 mA cm^−2^ owing to its abundant active sites, which are required for reducing oxygen at faster rates. Also smaller particle size (250 nm) of (3.0)Co‐N/C^Δ^ is advantageous for better mass‐transport of oxygen to the active sites, resulting in a higher peak power density. Even under a lower back pressure (0.5 bar gauge), (3.0)Co‐N/C^Δ^ maintained a current density ≈50 mA cm^−2^ at 0.8V_iR‐free_, yielding a peak power density of ≈1 W cm^−2^ (Figure S15a, Supporting Information). Hydrogen‐air polarization curves of (3.0)Co‐N/C^Δ^, (3.0)Fe‐N/C^Δ^, and (0.9)Co‐N/C(transmet.) are compared in Figure 4b. At 0.8 V, the measured current density reached ≈25 mA cm^−2^, which is considerable greater as compared to the other reported Co‐N/C catalysts (Table S8, Supporting Information). The (3.0)Co‐N/C^Δ^ catalyst delivered a current density of 0.54 A cm^−2^ at 0.60 V, which is particularly high for a Co‐N/C catalyst at practically relevant operating voltage, and even higher than that of (3.0)Fe‐N/C^Δ^. While the (3.0)Co‐N/C^Δ^ and (0.9)Co‐N/C(transmet.) exhibited similar kinetic activity, their peak power densities varied significantly. By refining the synthetic approach, the peak power density increased from 0.27 W cm^−2^ for (0.9)Co‐N/C(transmet.) to 0.39 W cm^−2^ still at a relatively high voltage of ≈0.49 V for (3.0)Co‐N/C^Δ^ (Figure 4b). At practical fuel cell operating potentials (0.6–0.7 V) under H_2_‐air conditions, the (3.0)Co–N/C^Δ^ catalyst achieved a performance comparable to that of Fe–N/C, further highlighting the potential of Co–N/C. The Tafel slope under H_2_‐O_2_ and H_2_‐air conditions shown in Figure 4c,d are almost identical for both catalysts with an average value of ≈75 mV decade^−1^ measured over one order of magnitude in current density. Furthermore, a stability test conducted at 0.5 V under hydrogen‐air conditions (Figure S16, Supporting Information) revealed a decrease in current density of ≈58% within 25 h, indicating a need for further improvements. However, referencing previous studies on similar catalysts, the degradation could potentially be attributed to the demetallation of active sites.^[^ ^6^ ^]^

## 3 Conclusion

We developed a high‐performing Co‐N/C catalyst via a rapid aqueous synthesis of ZIF‐8 doped with cobalt. By carefully controlling the synthetic parameters and carbonization steps, a Co‐N/C catalyst loaded with 3 wt% atomically dispersed cobalt sites and a small primary particle size of 250 nm was obtained. Atomic resolution STEM coupled with EELS confirmed atomic dispersion of cobalt sites without any side phases and fitting of EXAFS profiles revealed a tetrapyridinic structure of Co‐N_4_ sites. A remarkably high electrochemically accessible active site density of 3.58 × 10^19^ sites g^−1^ was reached with the optimized (3.0)Co‐N/C^Δ^ catalyst that is highest Co‐N/Cs to the best of our knowledge. A linear relationship was found between metal loading and electrochemically accessible sites, suggesting that higher Co loadings increase active site density, which in turn leads to enhanced overall ORR performance. Despite Co‐N/C's lower TOF compared to Fe‐N/C, high site density significantly boosted overall ORR activity, rivalling that of the Fe‐N/C. Loaded with 3 wt% atomic cobalt sites, the (3.0)Co‐N/C^Δ^ catalyst delivered excellent ORR performance with a mass activity of 3.5 A g^−1^ at 0.8 V_RHE_ in RRDE studies. Furthermore, (3.0)Co‐N/C^Δ^ exhibited promising performance in PEMFCs, achieving peak power densities of 1.33 W cm^−2^ under hydrogen‐oxygen and 0.39 W cm^−2^ under hydrogen‐air conditions, respectively. It would appear that in a similar catalyst structure, and under fuel cell operational conditions, Co‐based catalysts can equal and even exceed the performance of Fe‐based catalysts, answering the central question posed at the beginning of this study.

## 4 Experimental Section

### Synthesis of Catalyst

4.416 g of 2‐methylimidazole (53.33 mol, 99% Sigma–Aldrich) was dissolved in 48 g of DI water. 2 g of Zn(NO_3_)_2_·6H_2_O (6.72 mmol, ≥ 99% Sigma‐Aldrich) and 79.9 mg of CoCl_2_·6H_2_O (0.336 mmol [the molar ratio of Zn: Co is 20: 1], 98% Sigma–Aldrich) were dissolved in 12 g of DI water. 10.5 mL of trimethylamine solution (33 wt% in ethanol, Acros Organics) was added into 2‐methylimidazole solution with stirring. Then, the solution of Zn(NO_3_)_2_ and CoCl_2_ was added to the 2‐methylimidazole solution. The reaction was stirred for 30 min at room temperature. The resulting solution was then centrifuged (40 g, including the weight of centrifuge tube) to separate the product, and three water washes were done. The purple powder (≈1.4 g) was dried in the oven at 110 °C overnight.

After the drying, the catalyst was heated to 900 °C for 1 h under Ar (99.999%, Zero Grade, BOC), and then the catalyst was leached with 2 M H_2_SO_4_ (95%, VWR) solution under reflux to remove the zinc. After the acid leaching, the catalyst was washed for 3 times with ultrapure water (MilliQ 18.2MΩ cm). The catalyst was dried under vacuum at 80 °C overnight. The resulting material was mixed with the equal amount of NH_4_Cl (99.5%, BHD Laboratory Supplies) and ground with pestle and mortar. Finally, the catalyst was activated to 907 °C for 1 h under 5% H_2_/N_2_ (BOC). Synthesis of the transmetallated Co and Fe catalysts is described in Note S1 (Supporting Information).

### Physical Characterization

For the atomic‐scale analysis of cobalt sites, a probe spherical aberration‐corrected scanning transmission electron microscope (Jeol ARM 200 CF) equipped with a cold‐field emission electron source was employed. 80 keV and a low beam current were utilised. High‐angle annular dark‐field scanning transmission electron microscopy (HAADF‐STEM) images were captured with collection half‐angles of 68–180 mrad at a 24 mrad probe convergence semi‐angle. The detection of carbon, nitrogen, oxygen, and cobalt was performed using a Gatan Quantum ER dual EELS system, while elemental mappings were acquired with the Jeol Centurio EDXS system equipped with a 100 mm^2^ silicon drift detector. X‐ray absorption spectroscopy, containing both near‐edge structure (XANES) and extended X‐ray absorption fine structure (EXAFS) at the Co K‐edge of Co‐N/C catalysts were collected at BAMline (BESSY‐II) operated by the Helmholtz‐Zentrum Berlin of Materials and Energy.^[^ ^51^ ^]^ The powder samples were placed between Kapton foils, and the measurements were performed in transmission with two ionization chambers filled with Nitrogen. The monochromatic incident X‐ray beam was scanned with a Double Crystal Monochromator (DCM) Si (111) with an intrinsic energy resolution of deltaE/E = 2 × 10^−4^. The measurement protocol was the following: 10 eV steps until 20 eV before the edge, followed by 0.25 eV steps until 20 eV above the edge, and 2 eV steps until 200 eV above the edge. From then on equidistant k‐steps were taken (every 0.06 Å) until 16 Å. The acquired spectra were extracted, calibrated, and normalized using the IFFEFIT software package containing ATHENA and ARTEMIS software.^[^ ^52^ ^]^ The Fourier Transformations are made in k‐space (between 1.5 and 12 Å^−1^) and the resulting R‐space (1.2–3.6 Å) was used for fitting with the model structures. X‐ray photoelectron spectroscopy (XPS) was performed on a Thermo Scientific K‐Alpha X‐ray Photoelectron Spectrometer system (Al K α, 1486.6 eV). Inductively coupled plasma mass spectrometry (ICP‐MS) was taken by Agilent ICP‐MS 7900. Nitrogen physisorption analysis was performed to calculate the Brunauer–Emmett–Teller (BET) surface area, pore size and pore volume by a Micromeritics Tristar II 3020 instrument. The powder catalysts were dried at 140 °C overnight under nitrogen gas before the measurement. High‐purity nitrogen gas (BIP plus‐X47S) was used during analysis and high‐purity helium gas was used for the free‐space measurement. Raman spectra were acquired using the Bruker Confocal Raman Microscope SENTERRA II, with a wavelength of 532 nm and a glass slide as the substrate. Dynamic vapor sorption (DVS) measurements were conducted using a Surface Measurement Systems DVS‐ET. DI water (MilliQ, 18.2 MΩ cm), isopropanol (≥ 99%, VWR), and heptane (≥99%, Sigma–Aldrich) served as sorbents for DVS measurement. Co‐N/C, Pt/C (TKK TEC10E50E),Vulcan, and hot‐pressed Co‐N/C ink, which contained 46 wt% of Nafion ionomer, were examined. Catalysts were preheated under nitrogen (200 Standard cubic centimeters per minute (sccm)) to remove absorbed water and oxygen. During the pretreatment, the temperature was initially set to 80 °C for 10 min, then ramped up to 150 °C over 30 min and held for 3 h. Subsequently, the temperature was reduced to 80 °C over 30 min and finally to 25 °C over 3 h. For the testing, partial pressures of 0, 20, 50, 80, 98, 80, 50, 20, and 0% were applied successively, with each pressure maintained for 6 h while recording mass and dm/dt. The incubator temperature was maintained at either 25 °C (all solvents) or 80 °C (water, for selective materials).

### Oxygen Reduction Reaction Performance in Half Cell

5 mg of catalyst, 1038.5 µL of isopropanol solution (1:1 volume ratio of isopropanol: H_2_O) and 54 µL Nafion (5wt% solution, Sigma‐Aldrich) was sonicated for at least 30 min to get a homogenous dispersed ink. A loading of 0.2 mg cm^−2^ was deposited on the glassy carbon disk of a Ring Rotating Disk Electrode (Pine Instruments, model AFE6R1AU with glassy carbon as a disk with a concentric gold ring and rotator model AFMSRCE).

A custom made three‐compartment electrochemical glass cell was used; sulfuric acid (VWR, 95.0%) and ultrapure water (MilliQ 18.2MΩcm) were used to prepare 0.5 M electrolyte. A reversible hydrogen electrode (RHE) served as the reference electrode, connected to the electrochemical cell through a Luggin–Haber capillary. The counter electrode employed was a glassy carbon rod. Ultrapure oxygen and nitrogen gases (BIP plus, Air products) were utilized during the measurements. All measurements were conducted under a consistent rotation speed of 1600 rpm unless otherwise specified, and were monitored using the potentiostat (Autolab, model PGSTAT20). The oxygen reduction reaction (ORR) polarization was performed using linear sweep voltammetry (LSV), ranging from 1.00 V_RHE_ to 0.00 V_RHE_, with a scan rate of 5 mVs^−1^. Additionally, the gold ring was held at 1.50 V_RHE_ and the current measured was used to calculate the yield of H_2_O_2_ by Equation (1)

(1) $$H_{2}O_{2}\;\left(\%\right)=\frac{2I_{r}/N}{I_{d}+I_{r}/N}\;\times 100\%$$

where I_r_ is the current recorded by the gold ring, I_d_ is the disk current measured by glassy carbon, and N stands for the collection efficiency of the RRDE. Subsequently, the nitrogen background was measured by LSV under N_2_‐satutrated electrolyte within the same potential range and same scan rate as the ORR. Ohmic resistances were determined by electrochemical impedance spectroscopy (EIS) for the iR composition.

### Stability of Oxygen Reduction Reaction in Half Cell

A loading of 0.8 mg cm^−2^ was deposited on the RRDE, maintaining the same ORR performance measurement setup. All LSVs were acquired from 1.00 V_RHE_ to 0.00 V_RHE_, and cyclic voltammetry (CV) were conducted between 0.60 V_RHE_ and 1.00 V_RHE_ utilizing a scan rate of 5 mV s^−1^ for both techniques. To correct for background currents, a nitrogen‐saturated LSV was initially recorded. Subsequently, an oxygen‐saturated LSV was performed with EIS for iR correction. This was followed by 3000 cycles of CV. Sequentially, another LSV with EIS and 2000 cycles of CV were conducted. The stability test concluded with a final LSV with EIS.

### Determination of Site Density and Turnover Frequency

The detailed procedure of nitrite stripping follows that of the previous work.^[^ ^6^, ^11^, ^53^, ^54^ ^]^ Similar with the ORR measurement, a loading of 0.2 mg cm^−2^ was deposited on the glassy carbon disk of a Rotating Disk Electrode (with a mirror‐polished glassy carbon disk and rotator model AFMSRCE). A specially crafted three‐compartment electrochemical glass cell was employed in this study. The experimental setup included a glassy carbon counter electrode and a saturated calomel reference electrode (Sentek). An electrolyte solution of 0.5 m acetate buffer with a pH of 5.2 was prepared using sodium acetate (99%, Sigma‐Aldrich), glacial acetic acid (AnalaR Normapur, VWR), and ultrapure water (MilliQ 18.2MΩ cm). The pH of the electrolyte was confirmed by a pH meter (Thermo Scientific, Orion Versastar). Electrochemical measurements were conducted using a potentiostat (Autolab, model PGSTAT20) to apply the potential, while maintaining a rotating speed of 1600 rpm. Ultrapure nitrogen and oxygen (BIP, Air products) were utilized throughout the experiment. The use of a current integrator was essential to ensure the recording of all stripping charges, particularly as the reduction of nitrite occurred on the catalyst's surface.

### Proton‐Exchange Membrane Fuel Cell Tests

For a single‐cell PEMFC test, a Co‐N/C based cathode with a loading of ≈3.9 mg cm^−2^, a commercial anode with a loading of 0.4 mg_Pt_ cm^−2^ (Alfa Aesar, Johnson Matthey, Hydrogen Reformate/Cathode), and Nafion 211 membrane was used to construct the membrane electrode assemblies (MEAs).

20 mg of (3.0)Co‐N/C^Δ^ and 3.5 mg of Vulcan XC72R, which was used to enhance electrical conductivity were mixed with 260 mg of ultrapure water and 540 mg of isopropanol, and the solution was sonicated for 30 min. 435 µL of 5 wt% Nafion solution was added to the ink and sonicated for 1 h to make homogeneous dispersion. Then the ink was drop‐cast onto a 5 cm^2^ gas diffusion layer (GDL, 29 BC, SGL). The catalyst‐coated electrode was dried at 70 °C under vacuum for 30 min and then hot pressed with the commercial anode and Nafion membrane at 130 °C for 3 min under an applied pressure of 2.2 bar.

The geometric area of the MEAs was 5 cm^2^ and was installed in a serpentine type flow field (1 mm channel; 1 mm lands) single cell. Glass‐reinforced PTFE gaskets (Tygafluor) were employed to regulate MEA compression, ensuring it ranged between 20% and 25%. Fuel cell assessments were conducted at 80 °C by feeding hydrogen to the anode and oxygen/air to the cathode. All gases were fully humidified (100% RH), maintaining a pressure of 1 bar gauge on both sides unless stated. During H_2_‐O_2_ tests, the flow rates of both gases were sustained at 200 sccm. For H_2_‐air testing, flow rates of 300 sccm for hydrogen and 1000 sccm for air were employed. Both hydrogen and oxygen gases used were ultrapure grade standards (BIP Plus, Air Products). Polarization curves were captured using an 850e Fuel Cell Test System (Scribner Associates) by stabilizing the cell voltage for 1 min at each point.

For the stability test, H_2_ and air were used. In the beginning, three polarisation curves were recorded with 300 sccm of H_2_ and 1000 sccm of air. Then, the potential was held while current was recorded for 25 h, and the flow rates of H_2_ and air were 200 and 500 sccm, respectively. Finally, three polarisation curves were measured again with 300 sccm of H_2_ and 1000 sccm of air.

## Conflict of Interest

The authors declare no conflict of interest.

## Supporting information



Supporting Information

## Data Availability

The experimental data within the figures of this paper will be available for download from https://doi.org/10.5281/zenodo.17288283.
