# Supplementary material for: Designing Co–N/C Cathode Catalysts with Dense Atomic Cobalt Sites for Enhanced PEMFC Performance
Source: Adv Sci (Weinh). 2025 Oct 15;13(2):e16060. doi: 10.1002/advs.202516060 (PMC12786296; doi:10.1002/advs.202516060)
Supplement: Supplementary file 1 — Supporting Information [file ADVS-13-e16060-s001.pdf]

Supporting Information

Designing Co–N/C Cathode Catalysts with Dense Atomic Cobalt Sites for Enhanced PEMFC Performance

Mengjun Gong [a], Asad Mehmood [b], Ana Guilherme Buzanich [c], Tim-Patrick Fellinger [b], Colleen Jackson [a,d], Junyi Cui [e], Goran Drazic [f] and Anthony Kucernak [a]*

[a] Department of Chemistry, Imperial College London, Molecular Sciences Research Hub, White City Campus, London, W12 0BZ, United Kingdom

[b] Division for Electrochemical Energy Materials, Bundesanstalt für Materialforschung und -prüfung (BAM), Unter den Eichen 44-46, 12203 Berlin, Germany

[c] Division for Structure Analysis, Bundesanstalt für Materialforschung und -prüfung (BAM), Richard-Willstätter-Strasse 11, 12489 Berlin, Germany

[d] current address: Johnson Matthey Technology Centre, Blounts Court Road, Sonning Common, Reading RG4 9NH, United Kingdom

[e] Department of Chemical Engineering, Imperial College London, South Kensington Campus, London, SW7 2BZ, United Kingdom

[f] Department of Materials Chemistry, National Institute of Chemistry, Hajdrihova 19, SI-1000 Ljubljana, Slovenia

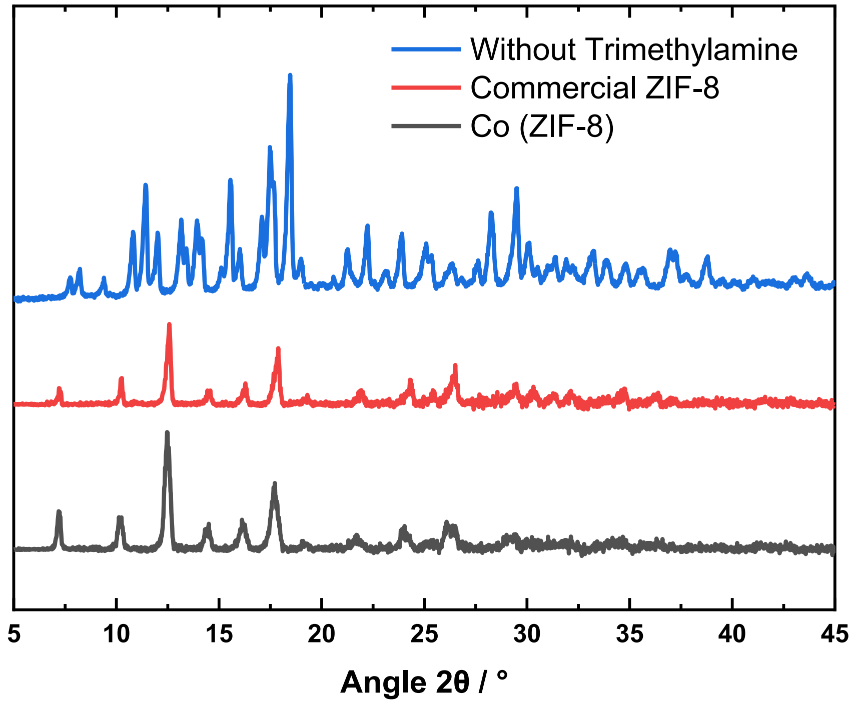

Figure S1. XRD of Co (ZIF-8), commercial ZIF-8 and without trimethylamine.

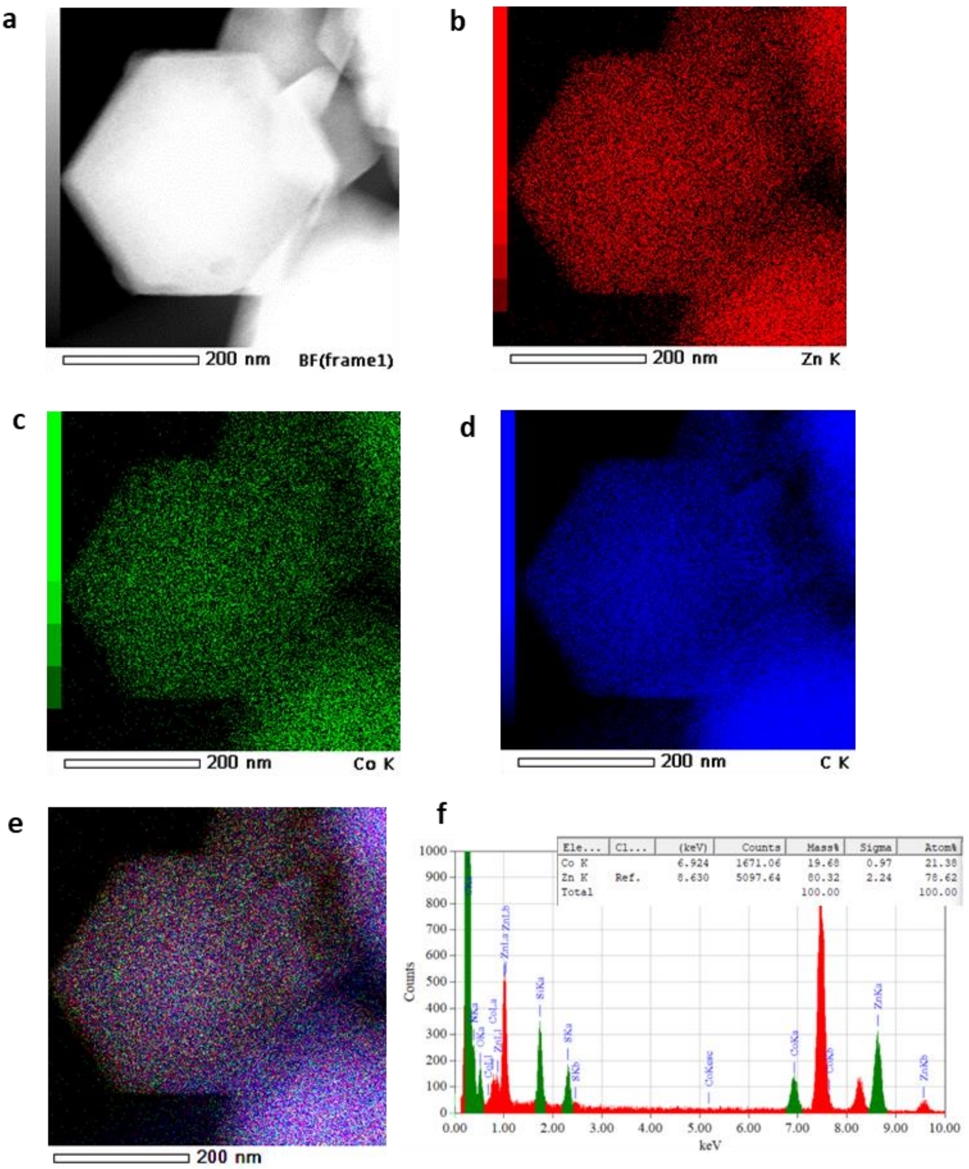

Figure S2. a) HAADF-STEM of (3.0)Co-N/C^{U}. b) c) d) EDXS elemental mapping of Zn, Co and C in Co-N/C^{U}. e) the overlap of the elemental mapping. f) EDX spectrum.

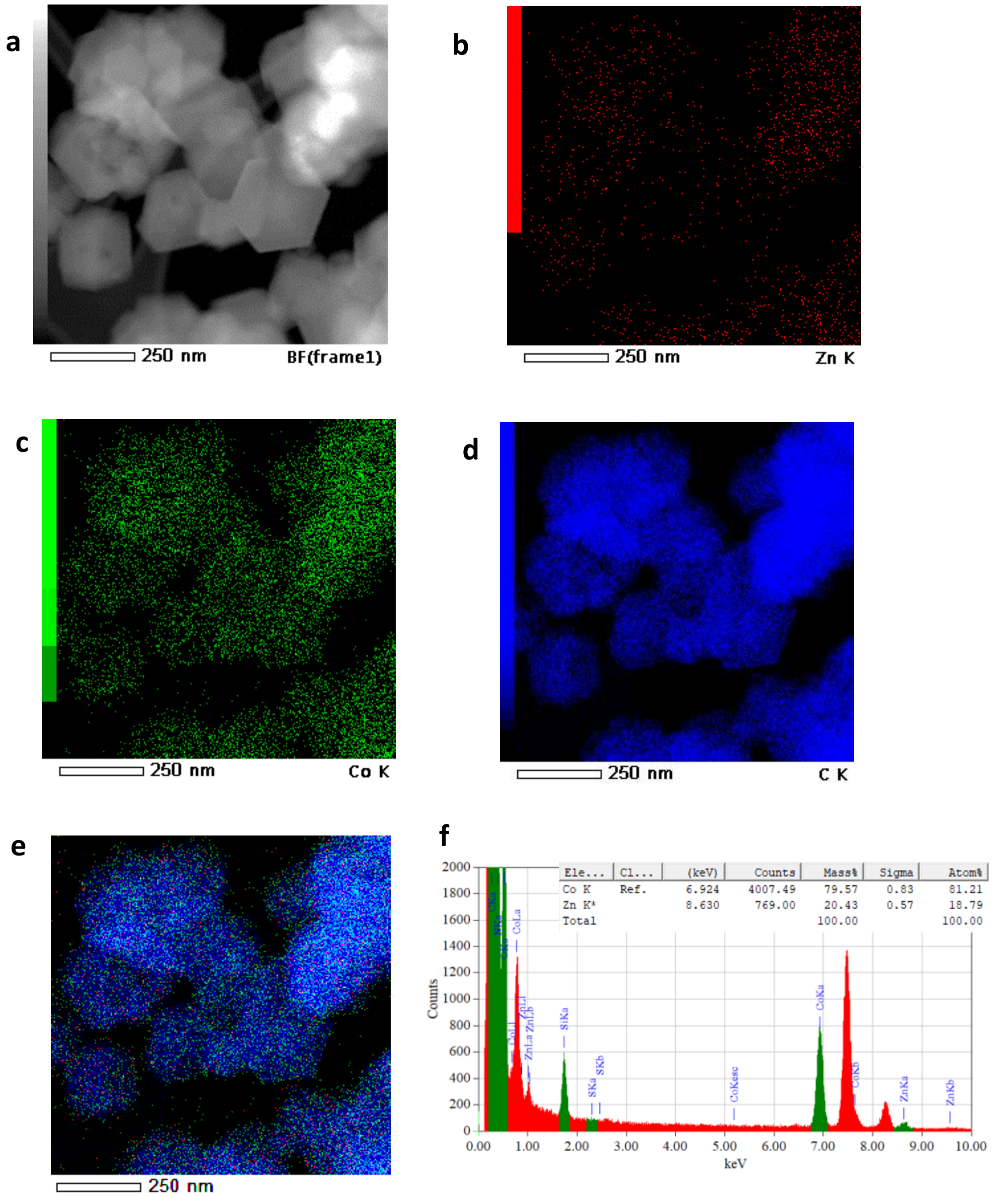

Figure S3. a) HAADF-STEM of (3.0)Co-N/C$^{\Delta}$. b) c) d) EDXS elemental mapping of Zn, Co and C in (3.0)Co-N/C$^{\Delta}$. e) the overlap of the elemental mapping. f) EDX spectrum.

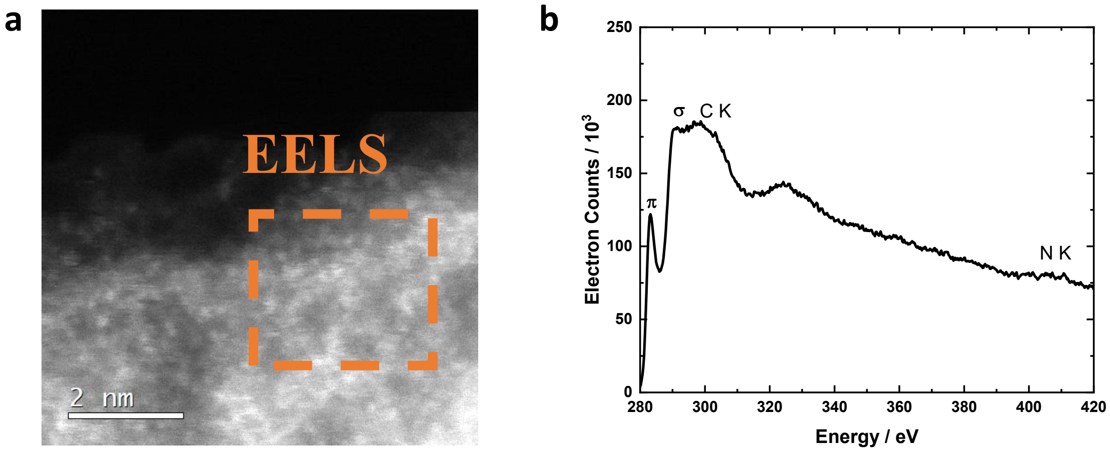

Figure S4. a) HAADF-STEM of (3.0)Co-N/C^{Δ} . b) EELS spectrum of C and N edge.

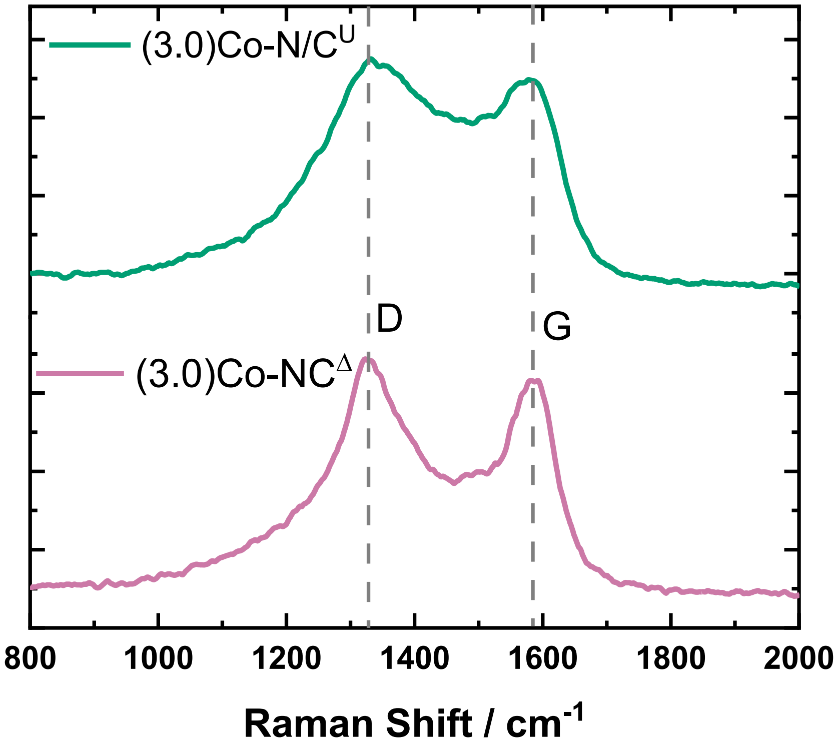

Figure S5. Raman spectroscopy of (3.0)Co-N/C^{U} and (3.0)Co-N/C$^{\Delta}$

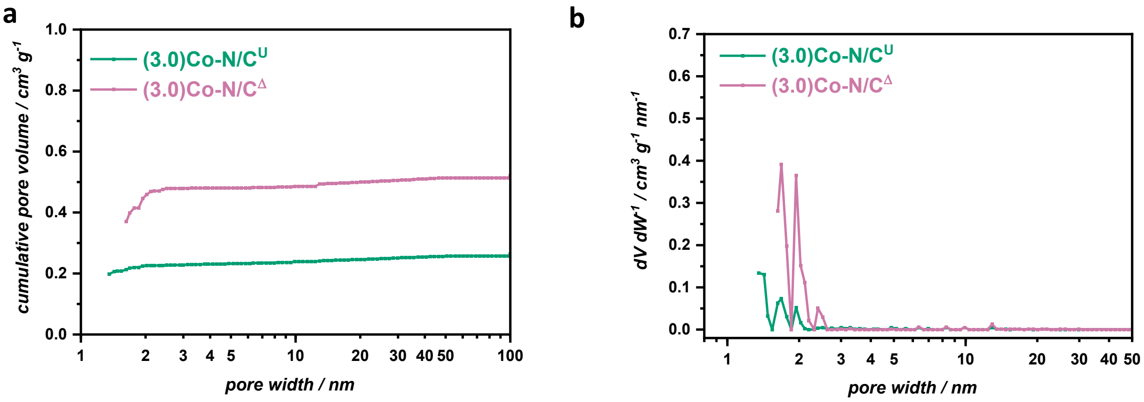

Figure S6. a) cumulative pore volume of (3.0)Co-N/C^U and (3.0)Co-N/C^Δ b) pore size distribution profiles of (3.0)Co-N/C^U and (3.0)Co-N/C^Δ

Table S1. Specific surface areas and micropore volumes of the Co-NC catalysts

Catalyst	BET surface/ m^2 g^{-1}	t-Plot Micropore Area/ m^2 g^{-1}	t-Plot Micropore Volume/ cm^3g^{-1}	BJH Adsorption cumulative volume of pores/ cm^3g^{-1}	BJH Desorption cumulative volume of pores/ cm^3g^{-1}
**(3.0)Co-N/C^U**	619.8	631.0	0.231	0.082	0.074
**(3.0)Co-N/C^Δ**	1,235.1	1,098.9	0.449	0.159	0.161
(0.5)Co-N/C (transmet.)[1]	1,100	887	0.373	0.233	0.236

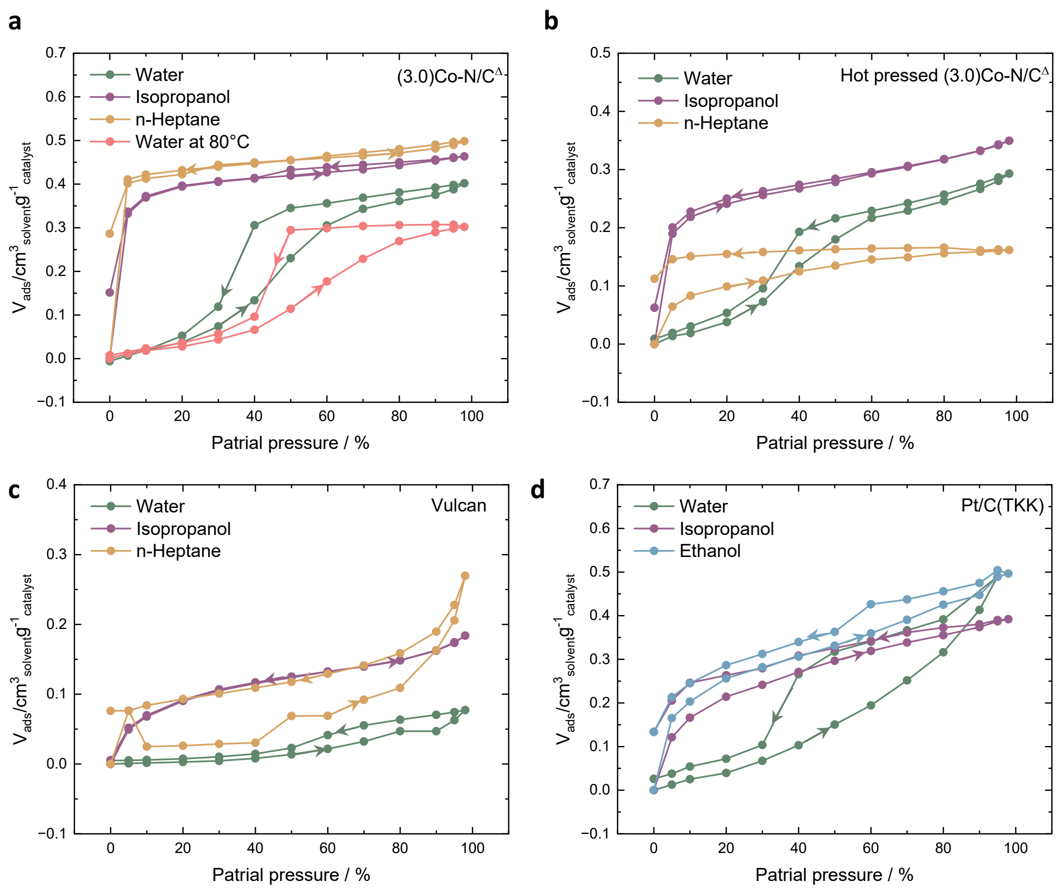

Figure S7. DVS isotherm of a) (3.0)Co-N/C$^{\Delta}$ b) hot-pressed (3.0)Co-N/C$^{\Delta}$ c) Vulcan and d) Pt/C (TKK TEC10E50E). Isotherms performed at 25°C apart from 80°C curve for water.

Table S2. Calculated parameter from DVS. Note that the values of the CCM/CCLs should be treated with caution as they are a heterogeneous system composed of two separate phases, and so the calculated parameters are a weighted composite of the multiple interfaces present. Pt/C catalyst TKK TEC10E50E

**Calculated parameters**	**Pt/C (TKK)**	**(3.0)Co-N/C$^{\Delta}$**	**Vulcan XC 72R**	**Pt/C TKK Hot pressed CCM**	**(3.0)Co-N/C$^{\Delta}$ Hot pressed CCL**
γ_s / mJ m^{-2}	120 ± 3	69.6 ± 28.7	61.3 ± 29.8	103 ± 1	67.9 ± 30.1
γ_s^d / mJ m^{-2}	21.3 ± 7.3	20.8 ± 9.8	16.7 ± 9.6	2.5 ± 0.3	16.6 ± 8.5
γ_s^p / mJ m^{-2}	99.2 ± 10.4	48.8 ± 35.3	44.6 ± 36.5	105 ± 1	51.3 ± 35.9
π_e / mJ m^{-2}	31.4 ± 9.0	8.8 ± 2.5	2.2 ± 0.7	6.1 ± 7.0	5.3 ± 3.5
W_{s-l} / mJ m^{-2}	107 ± 67	86.6 ± 60.3	80.1 ± 58.2	85 ± 65	83.1 ± 61.5

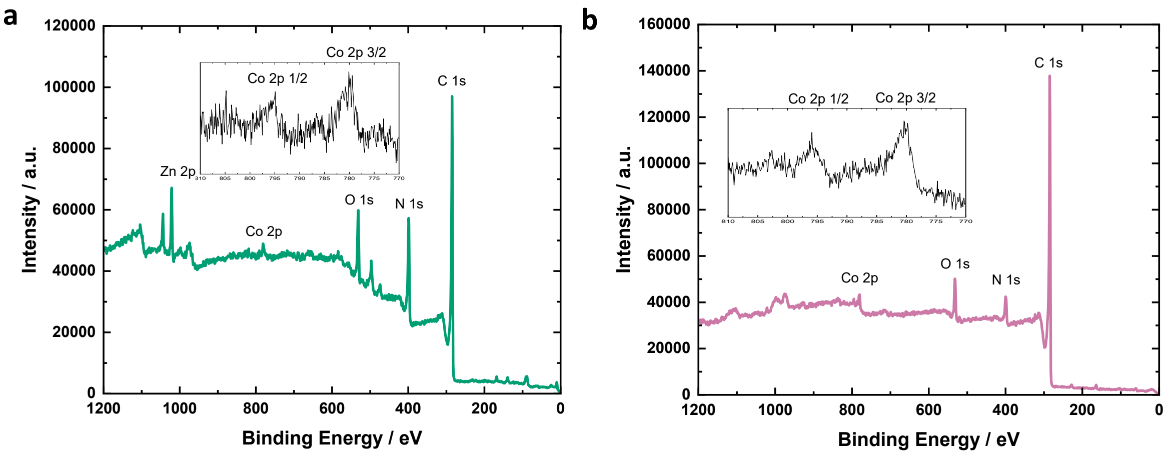

Figure S8. a) and b) survey XPS spectra of (3.0)Co-N/C^{U} and (3.0)Co-N/C^{Δ}

Table S3. Elemental composition of (3.0)Co-N/C^{U} and (3.0)Co-N/C^{Δ}. Determined by either XPS or ICP-MS

Catalyst	XPS / at%				ICP-MS/ wt%	
	N	O	Zn	Co	Zn	Co
**(3.0)Co-N/C^{U}**	17.2	6.28	0.58	0.15	8.6	1.0
**(3.0)Co-N/C^{Δ}**	5.59	4.38	/	0.23	3.1	3.0
(0.5)Co-N/C (transmet.) [1]	4.47	4.05	0.17	0.09	2.3	0.54

Table S4. Relative abundance of different nitrogen sites in (3.0)Co-N/C^{U} and (3.0)Co-N/C^{Δ}

	Pyridinic N	Co-N	Pyrrolic N	Graphitic N	NO_X
**(3.0)Co-N/C^{U}**	52.1	13.83	22.95	7.06	4.06
**(3.0)Co-N/C^{Δ}**	40.92	10.74	34.56	6.92	6.86
(0.5)Co-N/C (transmet.) [1]	46.84	16.81	23.77	7.61	4.97

Table S5. Best-fit parameters obtained from the EXAFS analysis of (3.0)Co-N/C^{U} and (3.0)Co-N/C$^{\Delta}$. Results from Co K-edge EXAFS fitted parameters, including scattering path, coordination number (N), interatomic distance (R), inter atomic distance from the model (Reff), Debey Waller factor (σ^2), amplitude reduction factor (S_O^2), energy shift parameter (ΔE_0), and R-factor, are given below. Co K-edge first-shell EXAFS of (3.0)Co-N/C^{U} and (3.0)Co-N/C$^{\Delta}$ was fitted to $(H_2O)_2CoN_4C_{10}$. The Hanning window type, with dk = 1 is chosen to perform the Fourier transformation over the selected k-space range between 1.5 to 12 Å^{-1}. The fit to the model structure was performed between r = 1.2 and 3.6 Å.

$(H_2O)_2CoN_4C_{10}$	Path	N	R / Å	R_{eff} / Å	S_0^2	σ^2 / 10^{-3} Å	ΔE_0
**(3.0)Co-N/C^{U}**	Co-N	4	1.89	1.91	0.84	6.5	-6.2
	Co-O	2	2.38	2.38		19.9	
**(3.0)Co-N/C$^{\Delta}$**	Co-N	4	1.91	1.91		7.6	-1.1
	Co-O	2	2.47	2.38		12.1	

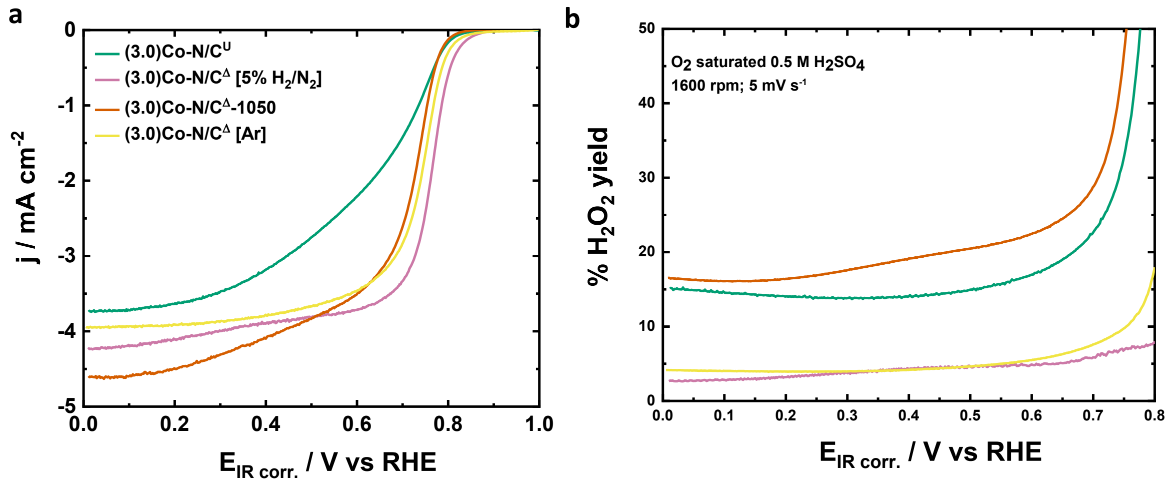

Figure S9. a) ORR polarisation of (3.0)Co-N/C^{U}, (3.0)Co-N/C$^{\Delta}$ [5% H_2/N_2], (3.0)Co-N/C$^{\Delta}$-1050 and (3.0)Co-N/C$^{\Delta}$ [Ar]with rotating rate 1,600 rpm in O_2-saturated 0.5 M H_2SO_4 electrolyte b) hydrogen peroxide yield of (3.0)Co-N/C^{U}, (3.0)Co-N/C$^{\Delta}$ [5% H_2/N_2], (3.0)Co-N/C$^{\Delta}$-1050 and (3.0)Co-N/C$^{\Delta}$ [Ar] Loading of catalyst: 0.2 mgcm^{-2}

Table S6. Comparison of Co-based single atom catalyst ORR activity

Catalyst	$E_{1/2}$ / V_{RHE}	Electrolyte	Rotation rate	Loading / mg cm^{-2}
Co-N-C (ZIF-8) [2]	0.70	0.5 M H_2SO_4	1600	
Co-Pyridinic N-C [3]	0.83	0.5 M H_2SO_4	1600	0.25
Co-N-C (ZIF-8) [4]	0.80	0.5 M H_2SO_4	900	0.8
Co(mIm)-NC(1.0) [5]	0.82	0.5 M H_2SO_4	900	0.6
ISAS-Co/HNCS [6]	0.77	0.5 M H_2SO_4	1600	0.5
Co-N/C(ZIF-8)[1]	0.70	0.5 M H_2SO_4	1600	0.2
Co–N–GA[7]	0.73	0.5 M H_2SO_4	1600	0.6
Fe/S_0-NC[8]	0.74	0.5 M H_2SO_4	1600	
Fe-N/C(CNRS)[9#]	0.76	0.5 M H_2SO_4	1600	0.8
Fe-N/C(PAJ)[9#]	0.78	0.5 M H_2SO_4	1600	0.8
Fe-N/C(UNM)[9#]	0.75	0.5 M H_2SO_4	1600	0.8
Fe-N/C(ICL)	0.73	0.5 M H_2SO_4	1600	0.2
(3.0)Co-N/C[Δ] (this work)	0.76	0.5 M H_2SO_4	1600	0.2

[#] The $E_{1/2}$ was obtained from the graph presented in the manuscript.

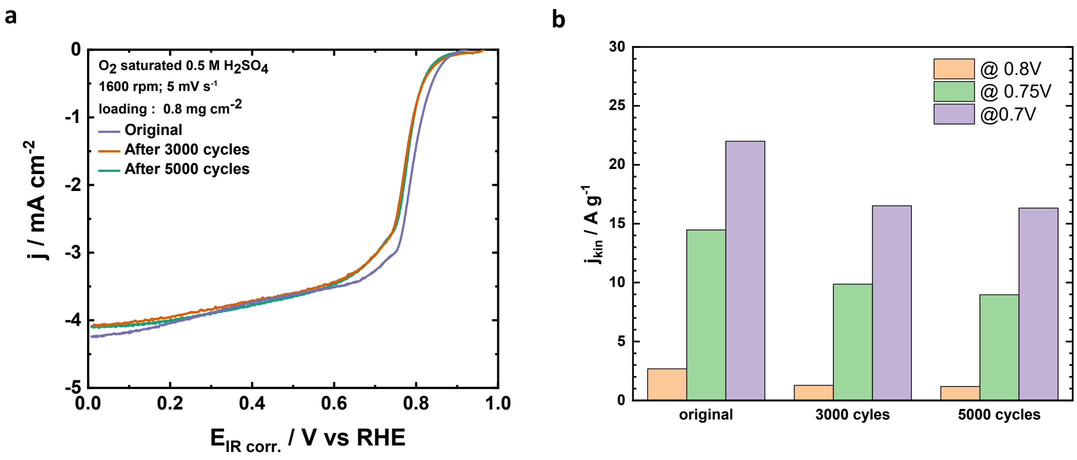

Figure S10. a) ORR polarisation curve of pristine, after 3,000 cycles and after 5,000 cycles in O_2-saturated 0.5 M H_2SO_4 electrolyte. b) the kinetic mass activity of these at 0.80 V_{RHE}, 0.75 V_{RHE} and 0.70 V_{RHE}. Loading of catalyst: 0.8 mg cm^{-2}.

ORR j_{kin} was calculated by: $j_{kin}(Ag^{-1}) = \frac{j_{lim@0.1V} \times j_{measured}}{j_{measured} - j_{lim@0.1V}}(mA\ cm^{-2}) \times \frac{1}{loading\ (mg\ cm^{-2})}$

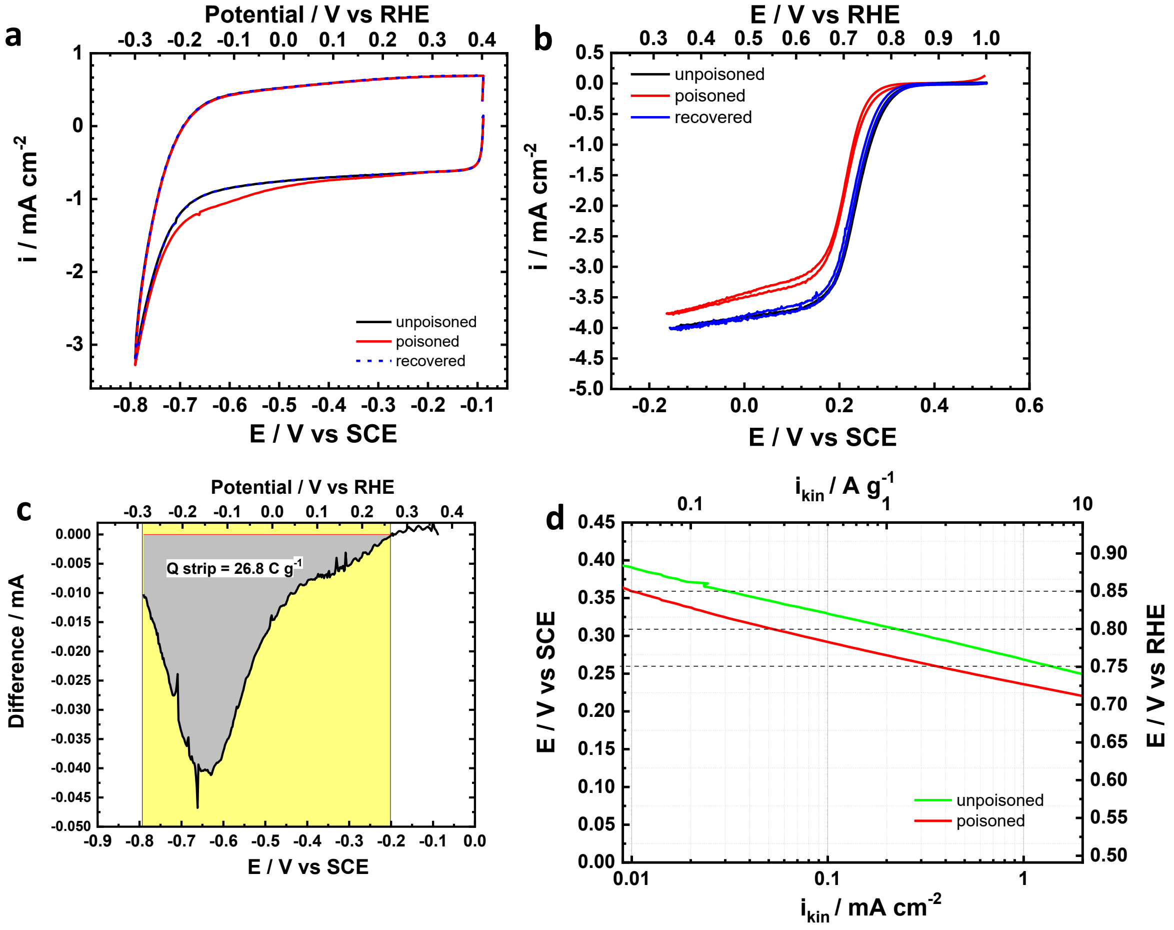

Figure S11. a) Cyclic voltammetry of nitrite stripping for unpoisoned, poisoned and recovered (3.0)Co-N/C$^{\Delta}$. b) ORR performance for unpoisoned, poisoned and recovered (3.0)Co-N/C$^{\Delta}$. c) current difference between poisoned and recovered. d) kinetic current densities from ORR measurement

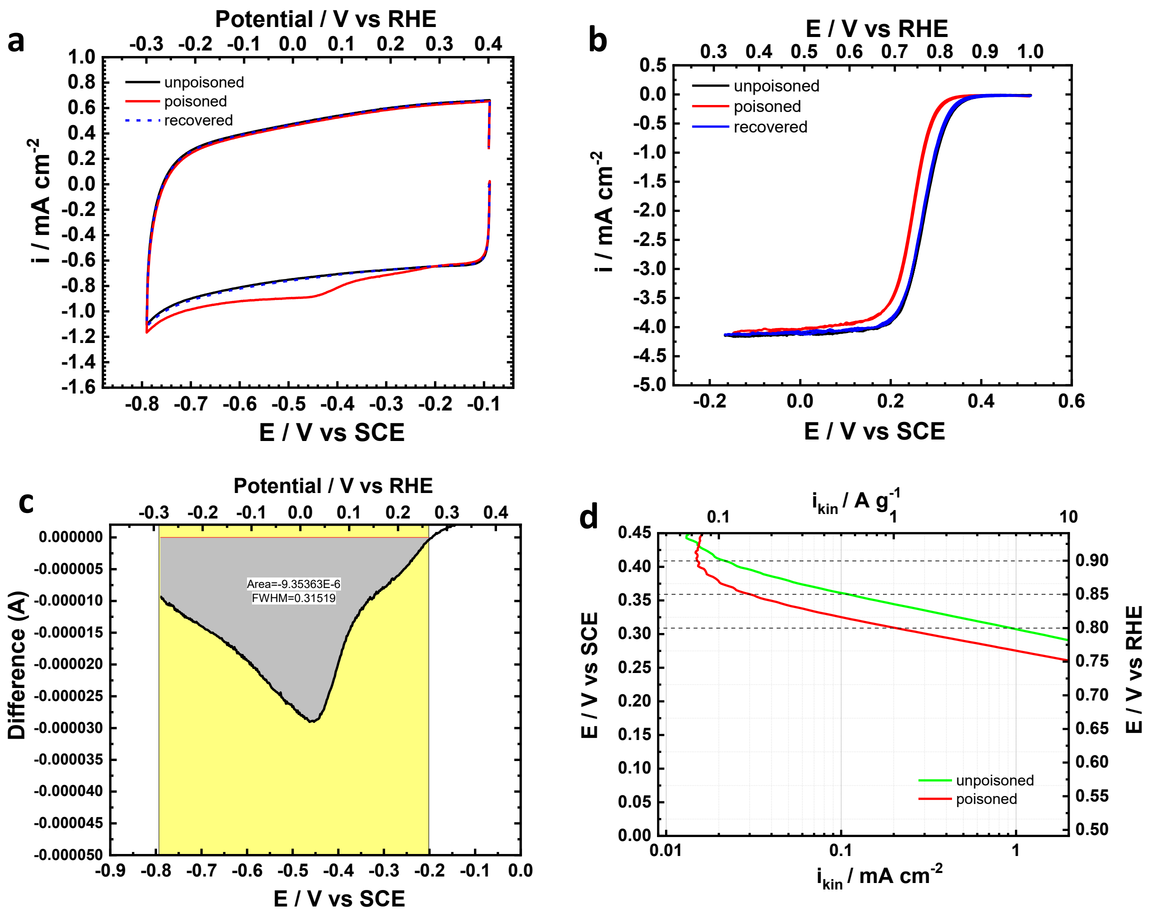

Figure S12. a) Cyclic voltammetry of nitrite stripping for unpoisoned, poisoned and recovered (3.0)Fe-N/C$^{\Delta}$. b) ORR performance for unpoisoned, poisoned and recovered (3.0)Fe-N/C$^{\Delta}$. c) current difference between poisoned and recovered. d) kinetic current densities from ORR measurement

Notes S1

(0.5)Co-N/C(transmet.)[1]: Commercial ZIF-8 (trade name Basolite Z1200, 100%) was utilised as a source of nitrogen and carbon. The pyrolysis process was conducted at 900 °C for 1 hour in a tube furnace (Carbolite) with a heating rate of 3 °C min^{-1} under a flowing atmosphere of Ar gas (99.99%, BIP plus-X47S, Air products). Subsequently, the resulting N/C material underwent leaching in 2 M H_2SO_4 (95%, VWR) overnight at 80 °C to eliminate Zn, which served as the templating metal, yielding a nitrogen-doped carbon (N/C) framework. In the subsequent step, cobalt ions were introduced into the N/C framework through transmetalation. To achieve this, 100 mg of N/C was dispersed in 100 mL of methanol (VWR) containing 25 mg of $CoCl_2 \cdot 6H_2O$. The mixture was reflux under stirring overnight, followed by thorough washing with deionised water (MilliQ 18.2 MΩ cm) and subsequent overnight washing in 0.5 M H_2SO_4 at room temperature to remove physically adsorbed non-precious metal ions. The resulting non-precious metal-coordinated catalyst underwent additional aqueous washing and was then dried in a vacuum oven overnight. Finally, a second heat-treatment step was carried out at 900 °C for 1 hour in a tube furnace under a flowing 5% H_2/N_2 (BOC) gas mixture, with a heating rate of 3 °C min^{-1}.

(0.8)Co-N/C$^{\Delta}$: prepared in the same way as the Co-N/C$^{\Delta}$; however, the amount of $CoCl_2 \cdot 6H_2O$ salt was 29.07 mg (0.122 mmol) instead of 79.9 mg. Therefore, the Co loading was lower than that (3.0)Co-N/C$^{\Delta}$.

(0.9)Co-N/C(transmet.) (tested in fuel cell): prepared following a procedure akin to that of the (0.5)Co-N/C(transmet.), outlined previously. A commercial ZIF-8 underwent pyrolysis at 900 °C for 1 hour. However, a deviation was introduced in the Zn removal process; instead of leaching with 2 M H_2SO_4 overnight at 80 °C, a reflux with 200 mL of 2 M H_2SO_4 was employed to eliminate the Zn content present in the catalyst and to great more sites for Co insertion. Subsequently, the N/C precursor underwent ball milling at 400 rpm for 2 h to reduce particle size. Next, 100 mg of ball-milled N/C catalyst was subjected to transmetalation with 50 mg of $CoCl_2 \cdot 6H_2O$ in 200 mL of methanol under reflux conditions. The resulting catalyst underwent dual water washes, followed by leaching with 0.5 M H_2SO_4 overnight at room temperature. This was succeeded by three additional rounds of water washing and subsequent vacuum drying. Finally, the catalyst was combined with DCDA (99%, Sigma-Aldrich) at a weight ratio of 2:1 and manually ground for 15 minutes before the activation step at 900 °C for 1 hour under a 5% H_2/N_2 gas mixture flow (BOC Ltd).

(3.0)Fe-N/C$^{\Delta}$: The Fe–N/C catalyst was prepared following the same procedure as (3.0)Co–N/C$^{\Delta}$, except that 66 mg of $FeCl_2 \cdot 4H_2O$ (0.336 mmol, 99%, Honeywell Fluka) was used in place of $CoCl_2 \cdot 6H_2O$ during the preparation of ZIF.

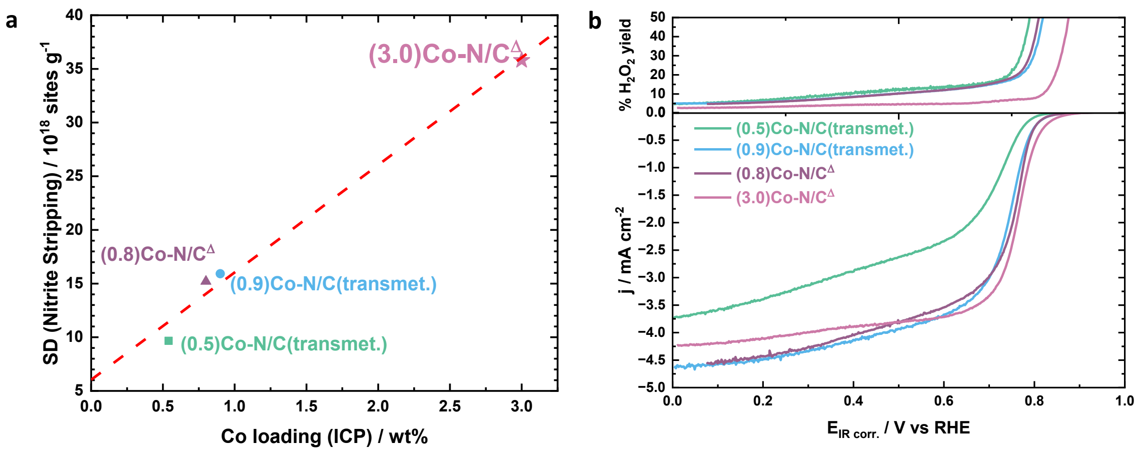

Figure S13. a) the relationship between Co loading tested from ICP-MS and site density calculated from nitrite stripping. b) ORR performance in O_2 saturated H_2SO_4. Loading of catalyst: 0.2 mg cm^{-2}.

Table S7. Electrochemical site density, ICP data and turnover frequency (TOF)

Name of Catalyst	Site Density/ x10^{19} sites g-1	Co loading (ICP) /wt%	% Utilisation	TOF $_{pH=0.3}$ @ 0.80 V
(0.5)Co-N/C (transmet.)[1]	0.967	0.54	17.52	0.28
(0.8)Co-N/C$^{\Delta}$	1.52	0.8	18.59	0.60
(0.9)Co-N/C (transmet.)	1.59	0.9	17.30	0.56
(3.0)Co-N/C$^{\Delta}$ (This work)	3.58	3.0	11.70	0.62

TOF was calculated by: $TOF_{pH=0.3}@0.8V\,(e\ site^{-1}s^{-1}) = \frac{j_{pH=0.3}@0.8V\ (Ag^{-1})}{SD\ (sites\ g^{-1})\times e(Ce^{-1})}$

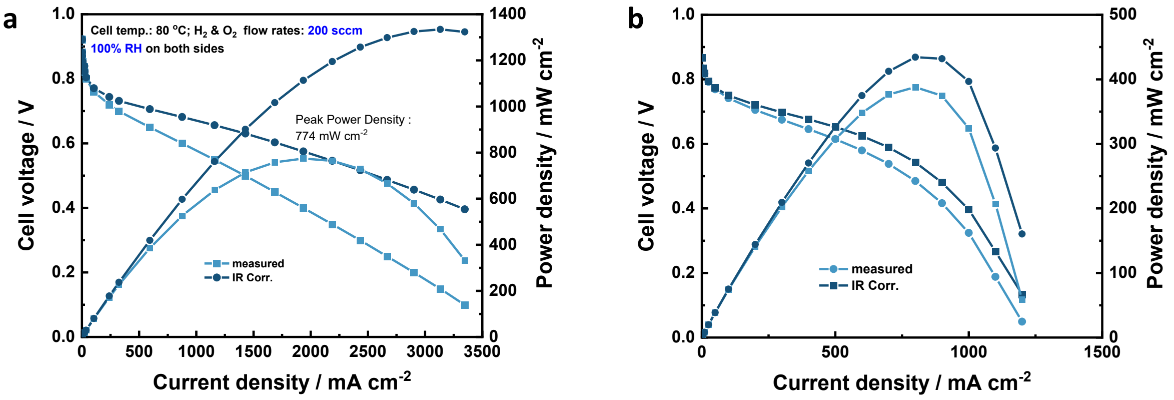

Figure S14. a) fuel cell polarisation curve under H_2-O_2 condition. b) fuel cell polarisation curve under H_2-Air condition. Test conditions: 5 cm^2 MEA consists of a 0.4 mg_{Pt} cm^{-2} Pt/C anode, a (3.0)Co-N/C^{Δ} catalyst cathode and a Nafion® 211 membrane. Measurement is done at 80 ˚C and 100% relative humidity. For H_2-O_2 test, the flow rates are 200 sccm for both sides. For H_2-air test, the flow rates of H_2 and air are 300 and 1,000 sccm respectively. 1 bar gauge pressure on both sides.

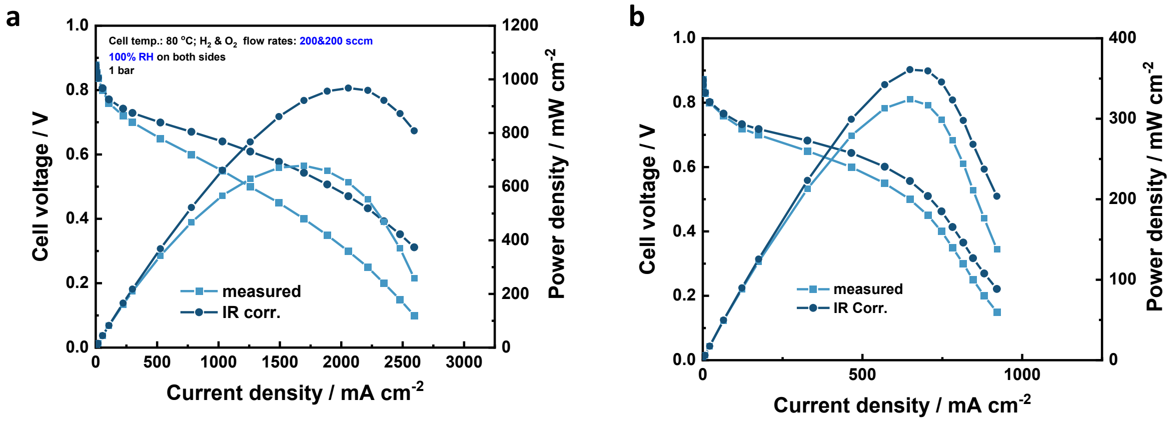

Figure S15. a) fuel cell polarisation curve under H_2-O_2 condition. b) fuel cell polarisation curve under H_2-Air condition. Test conditions: 5 cm^2 MEA consists of a 0.4 mg_{Pt} cm^{-2} Pt/C anode, a (3.0)Co-N/C^{Δ} catalyst cathode and a Nafion® 211 membrane. Measurement is done at 80 ˚C and 100% relative humidity. For H_2-O_2 test, the flow rates are 200 sccm for both sides. For H_2-air test, the flow rates of H_2 and air are 300 and 1,000 sccm respectively. 0.5 bar gauge pressure on both sides.

Table S8. Literature comparison of fuel cell performance

Catalyst	H_2-O_2 condition			H_2-Air condition			Loading / mg cm^{-2}
	Peak power density / W cm^{-2}	j @ 0.8 V /A cm^{-2}	pressure	Peak power density / W cm^{-2}	j @ 0.8 V /A cm^{-2}	pressure	
Co(mIm)-NC(1.0)[11]	~0.64 >1.4 iR-free	~0.022 (0.9 V)	1 bar	~0.32 ~0.45iR-free	not reported	1 bar	5.8
20Co-NC-1100[4]	~0.56	not reported	30 psi (2.75 bar)	~0.28	not reported	30 psi (2.75 bar)	4
Co-N-C@F127[12]	~0.87 iR-free	0.03	1 bar	~0.28 iR-Free	not reported	1 bar	4
Co@SACo-N-C-10[13]	0.42	not reported	2 bar	~0.23	not reported	2 bar	4
1.6%CoNC-ArNH3[14]	0.44, 0.502 iR-free	not reported	1 bar	~0.305	not reported	2 bar	3
CoNC@KJ600[15]	0.92	not reported	2 bar	not reported	not reported	not reported	4
P(AA-AM)(5-1)-Co-N[16]	0.66	0.047 (0.6 V)	1 bar	0.28	0.068 (0.4 V)	1 bar	3.2
d-(CoNP/CoSA–N–C)[17]	1.21	0.011	2 bar	0.45	0.088 (0.7 V)	1 bar	4
(Mn) Mn-N-C-S[18]	not reported	not reported	2 bar	~0.50	0.037 (0.7 V)	2 bar	4
(Fe) C-FeZID-1.44-950[19]	0.775	1.1 (0.6V)	30 psi (2.75 bar)	436	0.065 (0.6V)	30 psi (2.75 bar)	1
(3.0)Co-N/C$^{\Delta}$ (this work)	~0.68 ~0.97 iR-free	~0.055	1 bar	~0.32 ~0.36 iR-free	~0.021	1 bar	3.9
(3.0)Co-N/C$^{\Delta}$ (this work)	~0.77 ~ 1.33 iR-free	~0.050	2 bar	~0.39 ~0. 43 iR-free	~0.025	2 bar	3.9

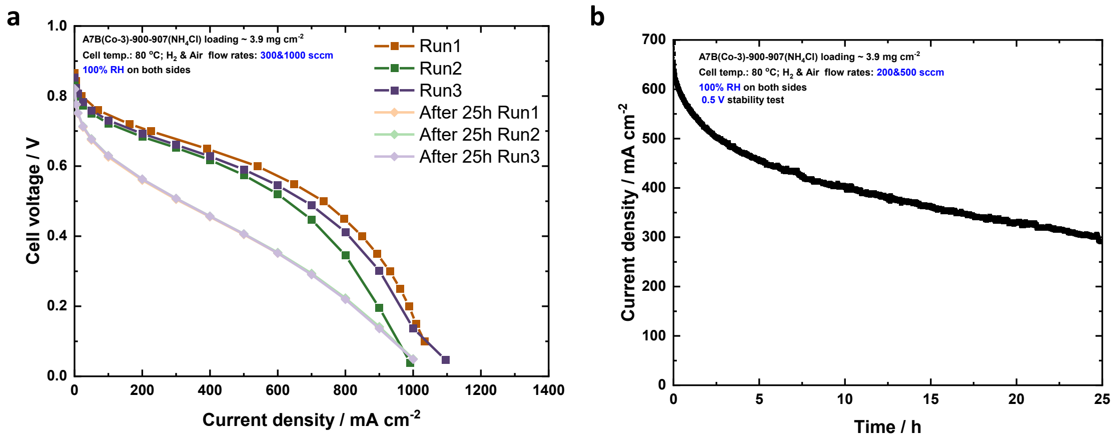

Figure S16. a) measured fuel cell polarisation curve under H_2-Air condition before stability test and after 25 h stability. b) current measured at 0.5V with time.

Test conditions: 5 cm^2 MEA consists of a 0.4 mg_{Pt} cm^{-2} Pt/C anode, a Co-N/C^{Δ} catalyst cathode with different type of carbon and a Nafion® 211 membrane. Measurement is done at 80 ˚C and 100% relative humidity. The flow rates of H_2 and air are 300 and 1,000 sccm respectively for polarisation curve. The flow rates for stability test are 200 and 500 sccm. 1 bar gauge pressure on both sides.

References

(1) Gong, M. J.; Mehmood, A.; Ali, B.; Nam, K. W.; Kucernak, A. Oxygen Reduction Reaction Activity in Non-Precious Single-Atom (M-N/C) Catalysts-Contribution of Metal and Carbon/Nitrogen Framework-Based Sites. *Acs Catal* **2023**, *13* (10), 6661-6674. DOI: 10.1021/acscatal.3c00356.
(2) Chao, G.; Zhang, Y.; Zhang, L.; Zong, W.; Zhang, N.; Xue, T.; Fan, W.; Liu, T.; Xie, Y. Nitrogen-coordinated single-atom catalysts with manganese and cobalt sites for acidic oxygen reduction. *Journal of Materials Chemistry A* **2022**, *10* (11), 5930-5936. DOI: 10.1039/d1ta08029f.
(3) Ha, Y.; Fei, B.; Yan, X.; Xu, H.; Chen, Z.; Shi, L.; Fu, M.; Xu, W.; Wu, R. Atomically Dispersed Co-Pyridinic N-C for Superior Oxygen Reduction Reaction. *Advanced Energy Materials* **2020**, *10* (46). DOI: 10.1002/aenm.202002592.
(4) Wang, X. X.; Cullen, D. A.; Pan, Y. T.; Hwang, S.; Wang, M.; Feng, Z.; Wang, J.; Engelhard, M. H.; Zhang, H.; He, Y.; et al. Nitrogen-Coordinated Single Cobalt Atom Catalysts for Oxygen Reduction in Proton Exchange Membrane Fuel Cells. *Adv Mater* **2018**, *30* (11). DOI: 10.1002/adma.201706758 From NLM PubMed-not-MEDLINE.
(5) Xie, X.; He, C.; Li, B.; He, Y.; Cullen, D. A.; Wegener, E. C.; Kropf, A. J.; Martinez, U.; Cheng, Y.; Engelhard, M. H.; et al. Performance enhancement and degradation mechanism identification of a single-atom Co–N–C catalyst for proton exchange membrane fuel cells. *Nat Catal* **2020**, *3* (12), 1044-1054. DOI: 10.1038/s41929-020-00546-1.
(6) Han, Y.; Wang, Y. G.; Chen, W.; Xu, R.; Zheng, L.; Zhang, J.; Luo, J.; Shen, R. A.; Zhu, Y.; Cheong, W. C.; et al. Hollow N-Doped Carbon Spheres with Isolated Cobalt Single Atomic Sites: Superior Electrocatalysts for Oxygen Reduction. *J Am Chem Soc* **2017**, *139* (48), 17269-17272. DOI: 10.1021/jacs.7b10194 From NLM PubMed-not-MEDLINE.
(7) Fu, X.; Choi, J. Y.; Zamani, P.; Jiang, G.; Hoque, M. A.; Hassan, F. M.; Chen, Z. Co-N Decorated Hierarchically Porous Graphene Aerogel for Efficient Oxygen Reduction Reaction in Acid. *ACS Appl Mater Interfaces* **2016**, *8* (10), 6488-6495. DOI: 10.1021/acsami.5b12746 From NLM PubMed-not-MEDLINE.
(8) Jin, H.; Zhao, X.; Liang, L.; Ji, P.; Liu, B.; Hu, C.; He, D.; Mu, S. Sulfate Ions Induced Concave Porous S-N Co-Doped Carbon Confined FeC(x) Nanoclusters with Fe-N(4) Sites for Efficient Oxygen Reduction in Alkaline and Acid Media. *Small* **2021**, *17* (29), e2101001. DOI: 10.1002/smll.202101001 From NLM PubMed-not-MEDLINE.
(9) Primbs, M.; Sun, Y.; Roy, A.; Malko, D.; Mehmood, A.; Sougrati, M.-T.; Blanchard, P.-Y.; Granozzi, G.; Kosmala, T.; Daniel, G.; et al. Establishing reactivity descriptors for platinum group metal (PGM)-free Fe–N–C catalysts for PEM fuel cells. *Energy & Environmental Science* **2020**, *13* (8), 2480-2500. DOI: 10.1039/d0ee01013h.
(10) Mehmood, A.; Gong, M.; Jaouen, F.; Roy, A.; Zitolo, A.; Khan, A.; Sougrati, M.-T.; Primbs, M.; Bonastre, A. M.; Fongalland, D.; et al. High loading of single atomic iron sites in Fe–NC oxygen reduction catalysts for proton exchange membrane fuel cells. *Nat Catal* **2022**, *5* (4), 311-323. DOI: 10.1038/s41929-022-00772-9.
(11) Xie, X. H.; He, C.; Li, B. Y.; He, Y. H.; Cullen, D. A.; Wegener, E. C.; Kropf, A. J.; Martinez, U.; Cheng, Y. W.; Engelhard, M. H.; et al. Performance enhancement and degradation mechanism identification of a single-atom Co-N-C catalyst for proton exchange membrane fuel cells. *Nat Catal* **2020**, *3* (12), 1044-1054. DOI: 10.1038/s41929-020-00546-1.
(12) He, Y.; Hwang, S.; Cullen, D. A.; Uddin, M. A.; Langhorst, L.; Li, B.; Karakalos, S.; Kropf, A. J.; Wegener, E. C.; Sokolowski, J.; et al. Highly active atomically dispersed CoN4 fuel cell cathode catalysts derived from surfactant-assisted MOFs: carbon-shell confinement strategy. *Energy & Environmental Science* **2019**, *12* (1), 250-260. DOI: 10.1039/c8ee02694g.
(13) Cheng, Q.; Han, S.; Mao, K.; Chen, C.; Yang, L.; Zou, Z.; Gu, M.; Hu, Z.; Yang, H. Co nanoparticle embedded in atomically-dispersed Co-N-C nanofibers for oxygen reduction with

high activity and remarkable durability. *Nano Energy* **2018**, *52*, 485-493. DOI: 10.1016/j.nanoen.2018.08.005.
(14) Chen, L. Y.; Liu, X. F.; Zheng, L. R.; Li, Y. C.; Guo, X.; Wan, X.; Liu, Q. T.; Shang, J. X.; Shui, J. L. Insights into the role of active site density in the fuel cell performance of Co-N-C catalysts. *Appl Catal B-Environ* **2019**, *256*. DOI: 10.1016/j.apcatb.2019.117849.
(15) Wang, R.; Zhang, P.; Wang, Y.; Wang, Y.; Zaghib, K.; Zhou, Z. ZIF-derived Co–N–C ORR catalyst with high performance in proton exchange membrane fuel cells. *Progress in Natural Science: Materials International* **2020**, *30* (6), 855-860. DOI: 10.1016/j.pnsc.2020.09.010.
(16) Miao, Z.; Xia, Y.; Liang, J.; Xie, L.; Chen, S.; Li, S.; Wang, H. L.; Hu, S.; Han, J.; Li, Q. Constructing Co-N-C Catalyst via a Double Crosslinking Hydrogel Strategy for Enhanced Oxygen Reduction Catalysis in Fuel Cells. *Small* **2021**, *17* (29), e2100735. DOI: 10.1002/smll.202100735 From NLM Medline.
(17) Cheng, X.; Yang, J.; Yan, W.; Han, Y.; Qu, X.; Yin, S.; Chen, C.; Ji, R.; Li, Y.; Li, G.; et al. Nano-geometric deformation and synergistic Co nanoparticles—Co-N4 composite sites for proton exchange membrane fuel cells. *Energy & Environmental Science* **2021**, *14* (11), 5958-5967. DOI: 10.1039/d1ee01715b.
(18) Guo, L.; Hwang, S.; Li, B.; Yang, F.; Wang, M.; Chen, M.; Yang, X.; Karakalos, S. G.; Cullen, D. A.; Feng, Z.; et al. Promoting Atomically Dispersed MnN(4) Sites via Sulfur Doping for Oxygen Reduction: Unveiling Intrinsic Activity and Degradation in Fuel Cells. *ACS Nano* **2021**, *15* (4), 6886-6899. DOI: 10.1021/acsnano.0c10637 From NLM PubMed-not-MEDLINE.
(19) Deng, Y.; Chi, B.; Li, J.; Wang, G.; Zheng, L.; Shi, X.; Cui, Z.; Du, L.; Liao, S.; Zang, K.; et al. Atomic Fe-Doped MOF-Derived Carbon Polyhedrons with High Active-Center Density and Ultra-High Performance toward PEM Fuel Cells. *Advanced Energy Materials* **2019**, *9* (13). DOI: 10.1002/aenm.201802856.
